# Parallel hippocampal-parietal circuits for self- and goal-oriented processing

**DOI:** 10.1073/pnas.2101743118

**Published:** 2021-08-17

**Authors:** Annie Zheng, David F. Montez, Scott Marek, Adrian W. Gilmore, Dillan J. Newbold, Timothy O. Laumann, Benjamin P. Kay, Nicole A. Seider, Andrew N. Van, Jacqueline M. Hampton, Dimitrios Alexopoulos, Bradley L. Schlaggar, Chad M. Sylvester, Deanna J. Greene, Joshua S. Shimony, Steven M. Nelson, Gagan S. Wig, Caterina Gratton, Kathleen B. McDermott, Marcus E. Raichle, Evan M. Gordon, Nico U. F. Dosenbach

**Affiliations:** ^a^Department of Neurology, Washington University School of Medicine, St. Louis, MO 63110;; ^b^Department of Psychiatry, Washington University School of Medicine, St. Louis, MO 63110;; ^c^Department of Psychological and Brain Sciences, Washington University in St. Louis, St. Louis, MO 63130;; ^d^Kennedy Krieger Institute, Baltimore, MD 21205;; ^e^Department of Neurology, Johns Hopkins University School of Medicine, Baltimore, MD 21205;; ^f^Department of Pediatrics, Johns Hopkins University School of Medicine, Baltimore, MD 21205;; ^g^Department of Cognitive Science, University of California, San Diego, CA 92093;; ^h^Mallinckrodt Institute of Radiology, Washington University School of Medicine, St. Louis, MO 63110;; ^i^Department of Pediatrics, University of Minnesota, Minneapolis, MN 55454;; ^j^Masonic Institute for the Developing Brain, University of Minnesota, Minneapolis, MN 55414;; ^k^Center for Vital Longevity, School of Behavioral and Brain Sciences, University of Texas at Dallas, Dallas, TX 75235;; ^l^Department of Psychiatry, University of Texas Southwestern Medical Center, Dallas, TX 75390;; ^m^Department of Psychology, Northwestern University, Evanston, IL 60208;; ^n^Department of Neurology, Northwestern University, Evanston, IL 60208;; ^o^Department of Biomedical Engineering, Washington University in St. Louis, St. Louis, MO 63130;; ^p^Department of Pediatrics, Washington University School of Medicine, St. Louis, MO 63110;; ^q^Program in Occupational Therapy, Washington University School of Medicine, St. Louis, MO 63110

**Keywords:** hippocampus, functional connectivity, brain networks, individual variability, resting state

## Abstract

The hippocampus is critically important for a diverse range of cognitive processes, such as episodic memory, prospective memory, affective processing, and spatial navigation. Using individual-specific precision functional mapping of resting-state functional MRI data, we found the anterior hippocampus (head and body) to be preferentially functionally connected to the default mode network (DMN), as expected. The hippocampal tail, however, was strongly preferentially functionally connected to the parietal memory network (PMN), which supports goal-oriented cognition and stimulus recognition. This anterior–posterior dichotomy of resting-state functional connectivity was well-matched by differences in task deactivations and anatomical segmentations of the hippocampus. Task deactivations were localized to the hippocampal head and body (DMN), relatively sparing the tail (PMN). The functional dichotomization of the hippocampus into anterior DMN-connected and posterior PMN-connected parcels suggests parallel but distinct circuits between the hippocampus and medial parietal cortex for self- versus goal-oriented processing.

## Significance

The finding that human hippocampal-cortical functional connectivity is nonunitary, separated along functional network borders (default mode network [DMN], self-oriented; parietal memory network [PMN], goal-oriented) in the anterior–posterior axis, raises various possibilities as to why this organization might be beneficial and could inform updates to current models of human hippocampal function, memory, and the self.

The hippocampus is critically important for a diverse range of cognitive processes, such as episodic and prospective memory, affective processing, and spatial navigation (1–7). The hippocampus’ diverse functions rely on its pattern of connectivity (8). Atypical cortico-hippocampal functional connectivity is associated with cognitive and affective deficits (9–12). A precise understanding of the functional organization of the hippocampus is crucial for understanding the neurobiology underlying hippocampally related diseases.

The hippocampus seems to exhibit functional heterogeneity along its longitudinal axis (anterior–posterior in humans; ventral–dorsal in rodents). Studies of the rodent hippocampus have demonstrated modular differentiation along its longitudinal axis in patterns of gene expression, function, and anatomical projections (2, 13, 14). The rodent ventral hippocampus (anterior in humans) plays a role in the modulation of stress and affect (2, 4), whereas the dorsal hippocampus (posterior in humans) is important for spatial navigation. Hippocampal place field representation sizes in rodent models also follow a ventral–dorsal gradient reflecting large-to-small spatial resolution (13, 14). The ventral hippocampus in rats is anatomically interconnected with the amygdala, temporal pole, and ventromedial prefrontal cortex (4, 15), while the dorsal hippocampus is connected with the anterior cingulate and retrosplenial cortex (4, 15).

In humans, evidence for structural differentiation between the anterior and posterior hippocampus is provided by age and Alzheimer’s disease–related hippocampal volume reduction differences (16) and diffusion tractography (17). Functional MRI (fMRI) research has suggested an anterior–posterior gradient in coarse-to-fine mnemonic spatiotemporal representations (18), such that anterior hippocampus supports schematics, while specific details associated with a given event are represented in posterior hippocampus (6, 7). Similarly, other studies have suggested anterior–posterior hippocampal differences in pattern completion (i.e., integrating indirectly related events) and pattern separation (i.e., discriminating between separate but similar events) (19).

Resting-state functional connectivity (RSFC) studies in humans have provided additional insights into the hippocampal connectivity that underlies hippocampus-mediated cognition. RSFC exploits the phenomenon that even in the absence of overt tasks, spatially separated but functionally related regions exhibit correlations in blood oxygen level–dependent (BOLD) signal (20–24). Group-averaged RSFC studies have found the hippocampus to be functionally connected to the default mode network (DMN) (25–28). The DMN is deactivated by attention-demanding tasks and thought to be important for self-referential processes, such as autobiographical memory, introspection, emotional processing, and motivation (26). Other group-averaged RSFC studies have reported the anterior hippocampus to be preferentially functionally connected to anterior parts of the DMN, while the posterior hippocampus was more strongly connected to the posterior DMN via the perirhinal and parahippocampal gyri (29–32).

Recent precision functional mapping studies have highlighted that RSFC group-averaging approaches obscure individual differences in network architecture in both the cortex and subcortical structures (33–41). The large amounts of RSFC data utilized (>300 min per subject) in precision functional mapping improve the signal-to-noise ratio and allow for the replicable detection of additional functional neuroanatomical detail in the cerebral cortex (34), cerebellum (33), basal ganglia, thalamus (35, 42), and amygdala (36). In a small, deep-lying structure like the hippocampus, group-averaging RSFC data may be even more problematic.

The medial parietal cortex is one of the main targets of hippocampal anatomical and functional connectivity (4, 15, 43–47) and was previously considered part of the DMN (25–28). The medial parietal cortex encompasses the swath of posterior midline neocortex between motor and visual regions. It includes the retrosplenial cortex, posterior cingulate, and precuneus (Brodmann Area 7, 23, 26, 29, 30, and 31). More recent studies revealed that parts of the medial parietal cortex belong to the parietal memory network (PMN) and the contextual association network (CAN) (23, 34, 39, 41, 48, 49). The PMN and CAN are immediately adjacent to the DMN in medial parietal cortex and therefore easily confounded in group-averaged data. The CAN (34, 41) corresponds to Braga et al.’s DMN subnetwork B (39). The identification of multiple different networks (DMN, PMN, CAN, and FPN [fronto-parietal network]) in medial parietal cortex reflects the ongoing recognition of novel networks, subnetworks, and organizational principles driven by precision functional mapping (23, 34, 39, 41, 50–52).

The DMN, PMN, and CAN are all thought to be important for memory. The DMN and PMN have been associated with different aspects of episodic memory processing. Autobiographical retrieval (i.e., memory over a lifetime) preferentially increases activity in the DMN, whereas memory for recently experienced events preferentially engages the PMN (27, 48, 53, 54). During explicit memory tasks, activity within the PMN decreases in response to novel stimuli but increases in response to familiar stimuli, such that increased activity seems to reflect attention to internal memory representations during retrieval (48, 55). The CAN processes associations between objects or places and their scenes (41, 56).

Here, we utilized precision functional mapping to examine individual-specific, hippocampal-cortical functional connectivity. We utilized both the Midnight Scan Club (MSC) dataset (*n* = 10 participants; 300 min. of resting-state fMRI data/subject) (34) and additional extremely highly sampled, higher-resolution resting-state fMRI data (2.6 mm isotropic voxels; 2,610 min; MSC06-Rep) from an independent dataset (57, 58). We generated individual-specific RSFC parcellations of the hippocampus, drawing on several advantages over group-averaging, including the following: (1) higher signal-to-noise ratio in deeper subcortical structures without blurring individual differences in network features and (2) more precise definition of individual-specific cortical functional network maps (i.e., DMN, PMN, CAN, and FPN).

## Results

**Fig. 1.**
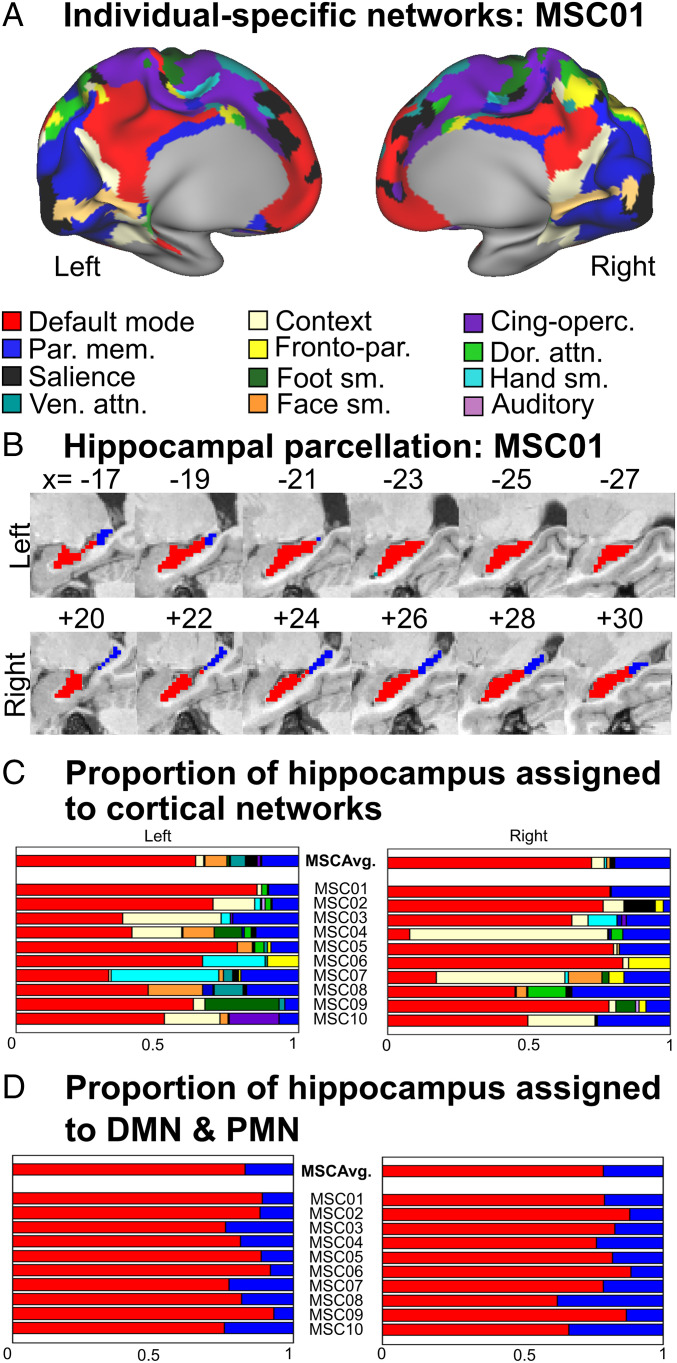
Hippocampal parcellation using a winner-take-all approach. (*A*) Resting-state networks in cortex, shown for exemplar subject (MSC01). Refer to *SI Appendix*, Fig. S1 for individual-specific resting-state networks of all subjects. (*B*) Winner-take-all parcellation of the hippocampus based on its functional connectivity to all cortical networks (MSC01), demonstrating a DMN-anterior and PMN-posterior organization. Parasagittal slices are shown. Refer to *SI Appendix*, Fig. S2 for individual-specific hippocampal parcellations of all subjects. (*C*) Quantification of the relative resting-state network representation in the left and right hippocampus for each MSC subject (1–10) and the MSC Average (*Top*), using a winner-take-all approach including all networks. (*D*) A two-network (DMN, PMN) winner-take-all approach. Refer to *SI Appendix*, Fig. S5 for the two-network winner-take-all parcellations for all subjects.

To characterize individual-specific hippocampal functional connectivity, we used a winner-take-all approach, such that each voxel was assigned to the cortical network with which it was most strongly functionally connected (*Materials and Methods*) (33, 35). We utilized 15 individual-specific networks generated from the Infomap community detection algorithm (34) (Fig. 1*A* and *SI Appendix*, Fig. S1).

### Anterior–Posterior Dichotomy in Hippocampus Functional Connectivity.

Individual-specific winner-take-all parcellations of the hippocampus revealed that the anterior hippocampus was most strongly functionally connected to the DMN in all individuals (Fig. 1*B* and *SI Appendix*, Fig. S2). Half of the subjects also exhibited connectivity of the anterior hippocampus to the CAN. In all subjects, the most posterior portion of the hippocampus (tail) was most strongly functionally connected to the PMN (Fig. 1*B* and *SI Appendix*, Fig. S2*A*). In MSC06, the lower-resolution data (4 mm; 300 min.) showed the posterior hippocampus to be most strongly functionally connected to the FPN. However, winner-take-all of the higher-resolution dataset (2.6 mm; 2,610 min; MSC06-Rep) also showed the posterior hippocampus to be most strongly connected to the PMN (*SI Appendix*, Fig. S3).

We quantified the proportion of the hippocampus preferentially connected to each cortical network in the winner-take-all analyses (Fig. 1*C*), which revealed that on average, 56% of the hippocampus was most strongly connected to the DMN, 13% to the CAN, 14% to the PMN, and 2% to the FPN.

Given that the winner-take-all parcellation scheme cannot account for more than one winning network within a voxel, we also considered the second strongest cortical connection for each voxel, as previously published (35). We found that functional connectivity to the FPN was second to the PMN in the posterior hippocampus (*SI Appendix*, Fig. S4). Similarly, the runner-up to the DMN in the anterior portion of the hippocampus was the CAN.

### Head/Body of Hippocampus Functionally Connected to DMN, while Tail Connects to PMN.

The winner-take-all analyses using all 15 functional networks showed differences in network organization between anterior (DMN) and posterior hippocampus (PMN). To clarify this anterior–posterior dichotomy, we next utilized a two-alternative (DMN versus PMN), forced-choice, winner-take-all approach. In all 10 MSC subjects (Fig. 1*D* and *SI Appendix*, Fig. S2) including higher-resolution 2.6-mm validation data (MSC06-Rep; *SI Appendix*, Fig. S3), we found a separation between the anterior/middle (DMN) and most posterior (PMN) hippocampus. We also found that the DMN was most strongly connected to the anterior ∼80% of the hippocampus (range: 62 to 88%), with the posterior ∼20% connecting to the PMN (range: 12 to 38%).

Supplemental analyses demonstrated that the functional connectivity strength of each individual-specific hippocampal parcel to its winner cortical network was at least z(r) ≥ 0.2 (*SI Appendix*, Figs. S6 and S7). *SI Appendix*, Fig. S8 visualizes the individual-specific functional connectivity of the DMN and PMN hippocampal parcels to the remaining networks.

**Fig. 2.**
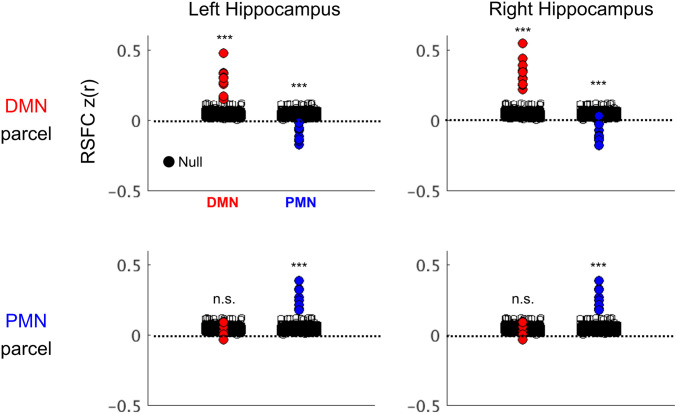
DMN and PMN parcels’ functional connectivity to cortical networks. Displayed is the mean RSFC, Fisher z-transformed correlations z(r), to the cortical DMN (red) and PMN (blue) for individual-specific winner-take-all–derived hippocampal DMN and PMN parcels. Black circles indicate the null distribution, generated from hippocampal winner-take-all parcellations between all possible network pairs; plotted are each generated parcels’ mean functional connectivity to its winner network. Although the null distribution for all participants is shown here, significance testing to demonstrate hippocampal parcels did not occur by chance and only occurred within subjects against the participant-specific null distribution. ***P < 0.001 for all subjects, n.s. *P* > 0.05.

The differences in network connectivity between anterior and posterior hippocampus allowed us to generate individual-specific DMN and PMN hippocampal parcels (Fig. 1*B*). Next, we verified that these parcels could not have been generated by chance (Fig. 2). Hence, we constructed participant-specific null distributions by conducting winner-take-all analyses on the hippocampus using all possible pairs of networks (DMN, PMN, and CAN; FPN excluded) and calculated the resulting parcels’ mean functional connectivity to the winner network (Fisher z-transformed correlations: z[r]). We found that the DMN and PMN parcels’ functional connectivity to their winning networks (DMN and PMN, respectively) was significantly greater than for any other possible two forced-choice winner-take-all combinations for each subject (*P* < 0.001 for all comparisons for all subjects). The DMN parcels (left and right) were strongly positively correlated with DMN in every subject but negatively correlated with the PMN (Fig. 2, *Top*). The PMN parcels were strongly positively correlated to the PMN and uncorrelated with the DMN (Fig. 2, *Bottom*).

We similarly tested the statistical significance of the anterior and posterior hippocampus’ preferential functional connectivity to CAN and FPN (*SI Appendix*, Figs. S4 and S8). We again computed the winner-take-all on the hippocampus using two network candidates (CAN and FPN) (*SI Appendix*, Fig. S9*A*). The functional connectivity of the resulting parcels was also significantly different from the null distribution (*SI Appendix*, Fig. S9*B*). The CAN–FPN division of the hippocampus strongly spatially overlapped with the DMN–PMN border (median Dice coefficient = 0.8; range = 0.4 to 0.9).

### Connectivity of Hippocampal Parcels Matches Individual-Specific Network Boundaries.

**Fig. 3.**
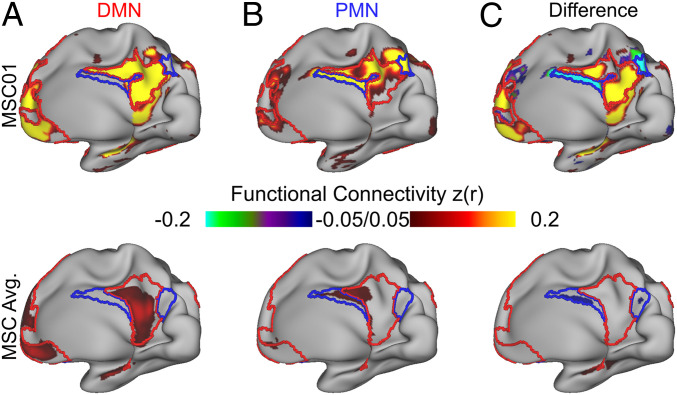
Functional connectivity of individual-specific hippocampal DMN and PMN parcels. (*A*) Functional connectivity of right hippocampal DMN parcel to cortex for exemplar subject (MSC01, *Top*) and MSC average (*Bottom*). (*B*) Functional connectivity of right hippocampal PMN parcel to cortex for exemplar subject (MSC01, *Top*) and MSC average (*Bottom*). (*C*) Difference between cortical connectivity of DMN and PMN parcels (DMN–PMN), showing that hippocampal functional connectivity respects individual-specific network borders. Warm colors represent greater DMN connectivity, and cool colors represent greater PMN connectivity. Functional connectivity values are Fisher z-transformed z(r). Refer to *SI Appendix*, Fig. S10 for all subjects.

To visualize the cortical functional connectivity of the individual-specific hippocampal parcels (anterior, DMN; posterior, PMN), we displayed it over the previously defined (34) individual-specific cortical functional network boundaries (Fig. 3 and *SI Appendix*, Fig. S10). Using the hippocampal DMN and PMN parcels as regions of interests, we generated seed maps (Fig. 3 *A* and *B*). Subtracting the individual-specific hippocampal seed maps (DMN parcel–PMN parcel) revealed sharp boundaries between the DMN and PMN in medial parietal cortex (Fig. 3*C*). Despite the DMN and PMN being immediately adjacent to one another in medial parietal cortex, differences in hippocampal-cortical functional connectivity between the anterior and posterior hippocampus retraced the boundaries of their respective cortical networks (DMN and PMN) in an individual-specific manner. In contrast, the difference map between the group-averaged DMN and PMN parcel seed maps did not show clear distinctions between the DMN and PMN networks (Fig. 3, *Bottom*).

### DMN–PMN Functional Connectivity Defines Functional Border in the Hippocampus.

**Fig. 4.**
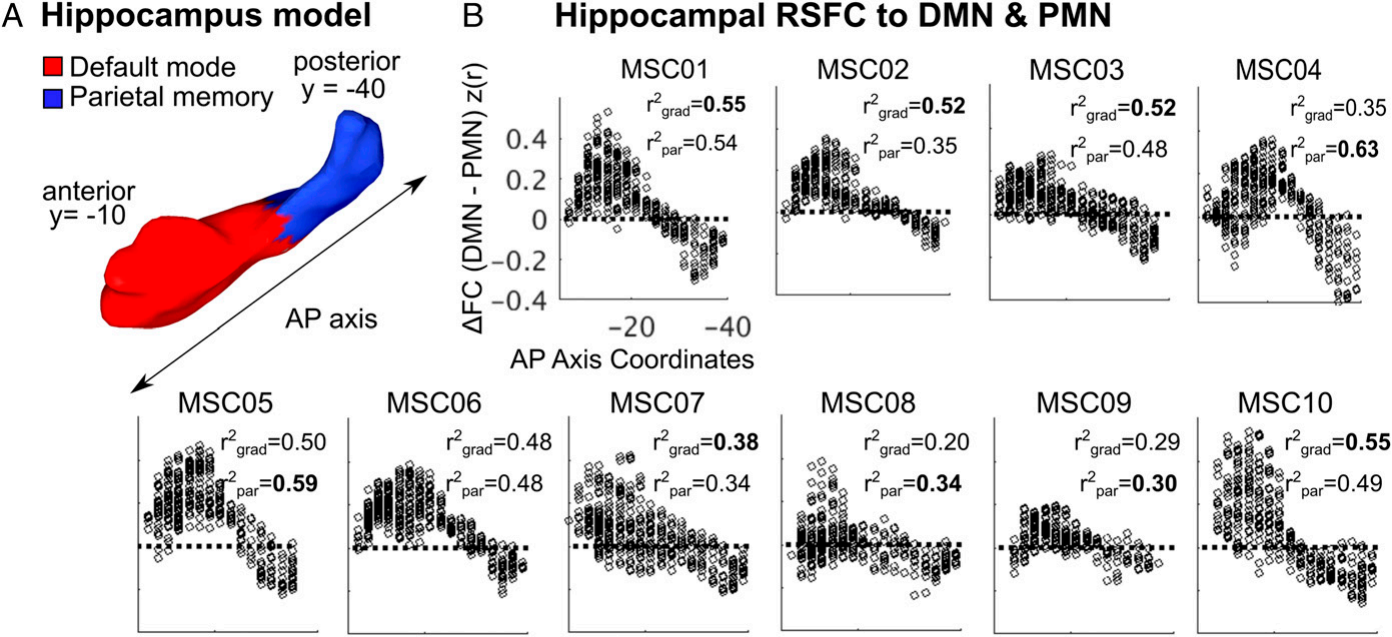
Hippocampal functional connectivity to DMN and PMN along the anterior–posterior axis. (*A*) Schematic of the hippocampus (MSC01) with the longitudinal (AP) axis drawn and the parcels outlined. For each subject, we determined each voxel’s position along the longitudinal axis in the hippocampus as well as its parcel identity. (*B*) Scatterplots depicting the pairwise differences in functional connectivity to DMN and PMN as a function of coordinate position along the longitudinal axis. The amount of variance in functional connectivity differences explained by the gradient (grad) and parcel (par) models are noted, suggesting equal variance explained and thereby both a gradient and parcel organization. Functional connectivity values z(r) are Fisher z-transformed.

To evaluate gradient versus parcel explanations of hippocampal organization, we tested whether the hippocampus’ functional connectivity was better explained by an anterior–posterior gradient or by network parcels using a one-way ANOVA (Fig. 4). For each hippocampal voxel, we calculated the difference between its correlation with the DMN and the PMN (ΔFunctional Connectivity [FC] = FC to DMN – FC to PMN; ANOVA: ΔFC ∼ longitudinal axis coordinates [gradient] or parcel identity). Across all subjects, both factors (${\text{r}}_{\text{par}}^{2}$ versus ${\text{r}}_{\text{grad}}^{2}$) explained roughly equal amounts of variance with some interindividual differences (Fig. 4). The r^2^ across the 10 MSC subjects for parcels (mean ${\text{r}}_{\text{par}}^{2}$ = 0.48) and gradient (mean ${\text{r}}_{\text{grad}}^{2}$ = 0.46) were similar. When replicating the gradient versus parcel analysis in the more highly sampled, higher–spatial resolution MSC06-Rep data, we found that parcel identity and gradient also explained similar amounts of variance (${\text{r}}_{\text{par}}^{2}$ = 0.77; ${\text{r}}_{\text{grad}}^{2}$ = 0.74) (*SI Appendix*, Fig. S3*D*). We also entered the gradient and parcel identity factors into a single analysis of covariance to calculate the variance explained by one factor after controlling for the other factor (*SI Appendix*, Table S1). We found that both factors simultaneously explained separate variance, demonstrating potentially superimposed gradient and parcel organization in the hippocampus.

Having established that parcels and gradients explain similar amounts of variance, we tested for a functional border between hippocampal DMN and PMN parcels using receiver–operator characteristic (ROC) analyses (*SI Appendix*, Fig. S11). We defined border voxels as adjacent to the other parcel within a two-voxel radius and calculated the connectivity similarity (to cortex) for all possible pairs of border voxels. We sorted the similarity values based on whether the pair of voxels belonged to the same or different parcels (*SI Appendix*, Fig. S11 histograms) and generated an ROC curve for each individual (*SI Appendix*, Fig. S11). We found that border voxels that belonged to the same hippocampal parcel (DMN or PMN) were significantly more similar than voxels that belonged to different parcels for every individual (Area Under the Curve = 0.64 to 0.88; *P* < 0.001). Thus, all 10 subjects had a discernible functional border between the DMN and PMN parcels.

### Anatomical Segmentation of Hippocampus Matches Functional Parcellation.

**Fig. 5.**
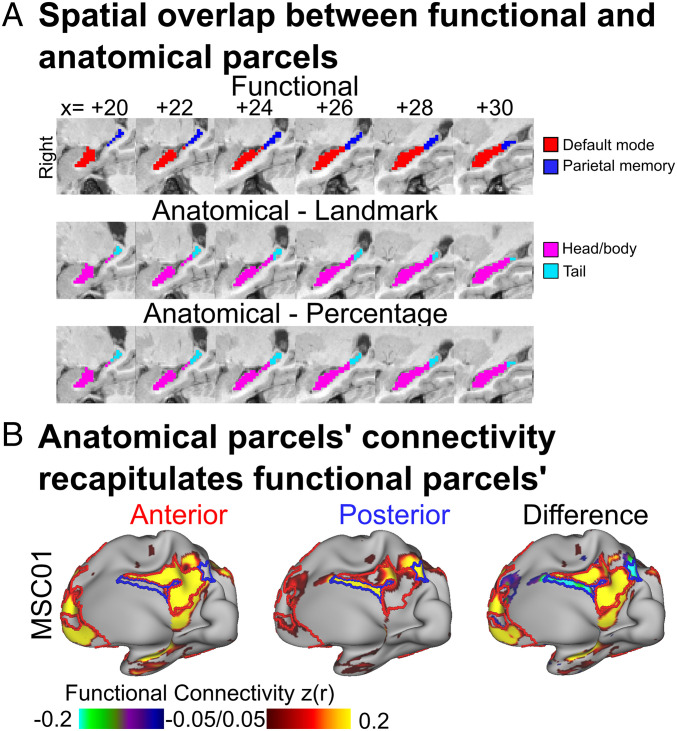
Functional connectivity of anatomically defined hippocampal segments (head/body versus tail). (*A*) The hippocampus was split into two segments, head/body and tail, based on either anatomical landmarks or a percentage-based approach (exemplar subject MSC01 shown). Refer to *SI Appendix*, Fig. S12 for landmark and percentage-based anatomical hippocampal parcellations for all subjects, demonstrating a similar anatomical segmentation. (*B*) Functional connectivity seed map for anatomical head/body (*Left*), tail (*Middle*) parcels, and the difference between the two (*Right*), for MSC01 as an exemplar, recapitulating functional parcels. Functional connectivity values z(r) are Fisher z-transformed. Refer to *SI Appendix*, Fig. S13 for all subjects.

Winner-take-all functional parcellation (Fig. 1) segmented the hippocampus into DMN and PMN parcels. To test whether the functional parcellations revealed by the winner-take-all approach mapped onto anatomical definitions of the hippocampal head/body and tail, we examined the spatial overlap between functional parcels and anatomical segments. We used anatomical landmarks (59) to select the coronal slice that demarcates the border between the body and tail of the hippocampus (*SI Appendix*, Fig. S12). We found a high degree of spatial overlap between anatomical segments and winner-take-all parcels (Dice coefficient median: 0.84, range: 0.74 to 0.92) across all subjects (Fig. 5*A*). We also used a percentage-based approach (Fig. 5*A*) for segmenting the tail (posterior 20%) from the head/body (anterior 80%) of the hippocampus (3), which yielded very similar segmentations to the landmark approach (Dice coefficient median: 0.92, range: 0.85 to 0.96).

Next, we tested whether anatomically defined hippocampal segments (head/body versus tail) replicated the functional connectivity differences observed with functionally defined parcels (DMN versus PMN; Fig. 3). This validation analysis also found that functional connectivity patterns of the hippocampal head/body and tail differed starkly (Fig. 5*B*). That is, the head and body of the hippocampus were strongly preferentially connected to the DMN, and the tail was strongly preferentially connected to the PMN. The anatomical hippocampal segments mapped onto the functionally defined parcels well enough to potentially serve as a proxy.

### Task-General Deactivations Specific to the DMN while Relatively Sparing the PMN.

**Fig. 6.**
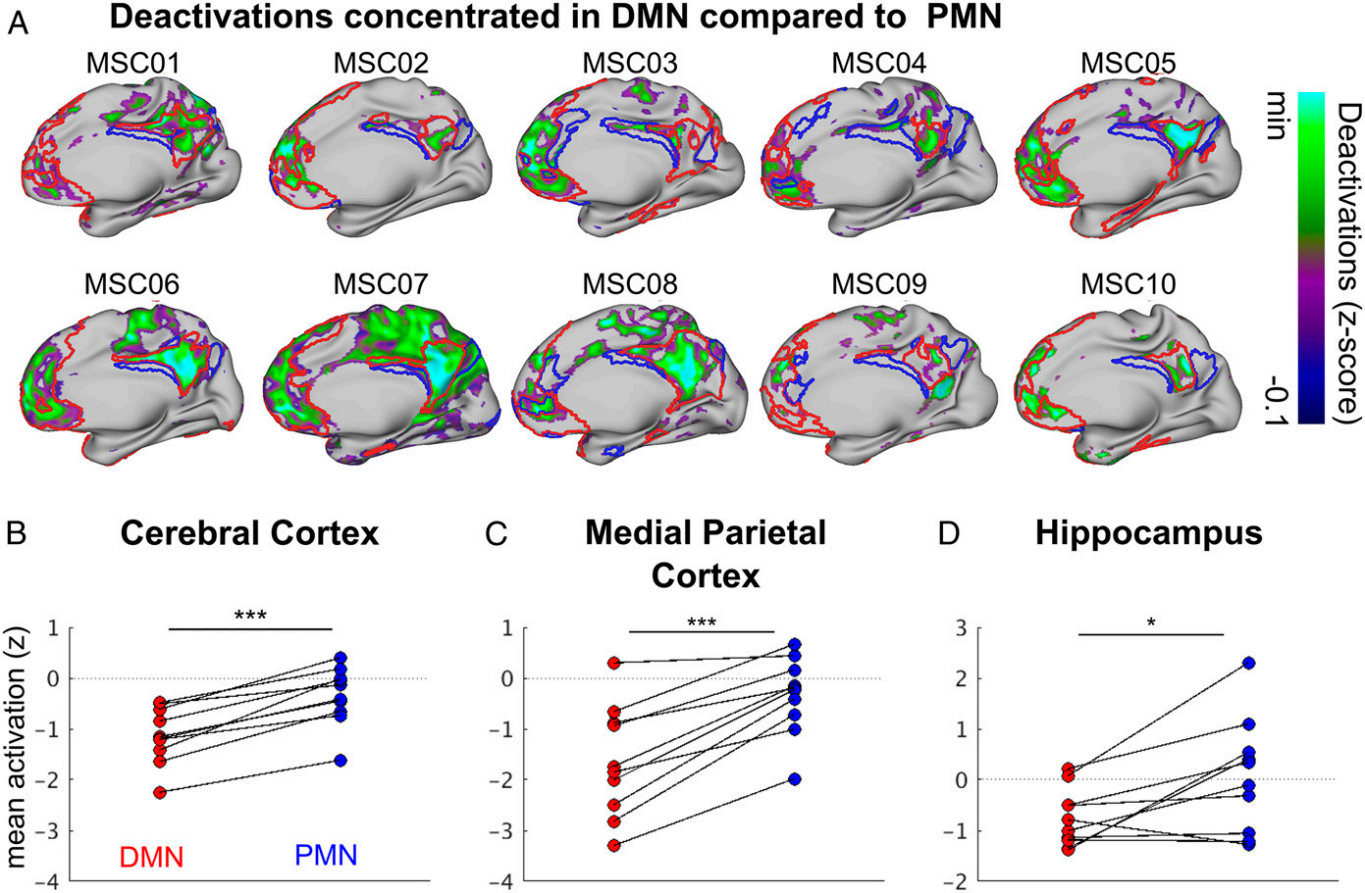
Task deactivations in DMN and PMN. (*A*) Whole-brain task-general fMRI signal decreases with RSFC defined network borders (DMN, red; PMN, blue) overlaid. Task deactivations (negative fMRI signal relative to baseline), are shown for all MSC subjects (right hemisphere, medial). Mean task activations (z-scores) in DMN and PMN were calculated for (*B*) all of cerebral cortex, (*C*) the medial parietal cortex, and (*D*) the hippocampus. On average, there were greater task-general activity decreases in the DMN compared to the PMN across the whole brain during the task state. ***P < 0.001; *P < 0.05

To validate the segregation of the DMN from the PMN, seen with resting-state and structural MRI, we next examined task-driven deactivations during spatial coherence and noun–verb discrimination tasks (mixed blocked event-related design) (34, 60). We found that robust task-driven decreases in activity were localized to the DMN, with less pronounced or no deactivations in the PMN (Fig. 6*A*). The task-general activity decreases, the DMN’s defining characteristic, were significantly greater for the DMN than for the PMN in the cortex as a whole (Fig. 6*B*; *P* < 0.001), as well as the DMN and PMN parcels in the medial parietal cortex (Fig. 6*C*; *P* < 0.001), and the hippocampus (Fig. 6*D*; *P* = 0.02). Thus, the dichotomization into parallel DMN and PMN hippocampal-parietal circuits was borne out by anatomy, functional connectivity, and task fMRI.

## Discussion

### Superimposition of Functional Parcels onto Hippocampal Gradients.

Prior studies have largely conceptualized the organization of the hippocampus along gradients—e.g., size of place field representations (13, 14) or spatiotemporal scale of mnemonic representations (6, 7). Using individual-specific precision functional mapping, we documented overlapping gradient and parcel organization along the hippocampal anterior–posterior axis (Fig. 4). We found the anterior ∼80% of the hippocampus (head and body) to be preferentially functionally connected to the DMN, with secondary connections to the CAN (Figs. 1 and 2). In contrast, the posterior hippocampus (tail) was preferentially functionally connected to the PMN, with secondary connections to the FPN. The DMN, CAN, PMN, and FPN are arranged as a topological ensemble, such that these networks are immediately adjacent to one another in both medial parietal cortex and hippocampus.

**Fig. 7.**
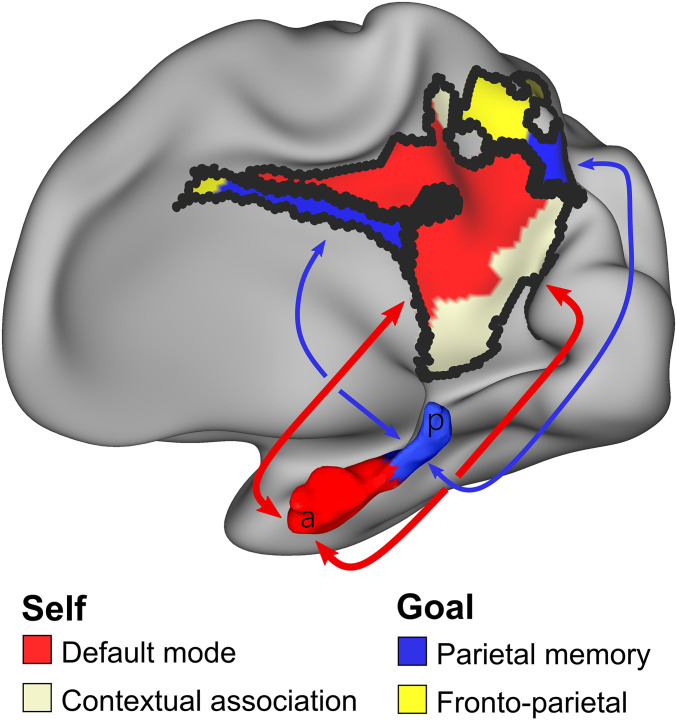
Schematic of parallel self- and goal-oriented circuits between the hippocampus and medial parietal cortex. Medial parietal cortex is the primary target of hippocampal functional connectivity, but connectivity is segregated by functional network. The bulk of the hippocampus (anterior) is functionally connected to the DMN (red) and CAN (pearl white) networks. The tail of the hippocampus is preferentially connected to the PMN (blue) and fFPN (yellow) networks. This functional connectivity dichotomy maps onto those parts of hippocampus and medial parietal cortex that deactivate during goal-oriented tasks (DMN and CAN) and those that do not (PMN and FPN). This functional organization suggests that human cognition can draw on two variants of hippocampal and medial parietal circuitry. The anterior circuit might support sequencing and navigating spacetime (68, 69) in the service of the self, while the posterior circuit might carry out very similar operations in the service-specific, goal-directed, attention-demanding tasks.

To test whether these results were affected by data amount, quality, or resolution, we used higher-resolution (2.6-mm) data from MSC06-Rep (2,610-min), which again showed a parcel and gradient organization. The parcellation of the hippocampus, based on resting-state fMRI data (Figs. 1 and 2), was validated by the localization of task-driven deactivations to DMN parcels while relatively sparing the PMN (Fig. 6). The dichotomization of the hippocampus into anterior and posterior functional circuits, validated with multiple modalities and analytical methods, gave rise to our dual-circuits model of hippocampal-cortical connectivity for self- and goal-oriented processing (Fig. 7).

### Anterior Hippocampus to Medial Parietal Cortex Circuit for Self-Oriented Processing.

Our results more precisely define the anterior 80% of the hippocampus as the hippocampal subregion that interacts with the cortical DMN, likely mediated by adjacent perirhinal and parahippocampal cortex (32, 61). The DMN is generally thought to mediate introspective, self-oriented types of cognition (62).

We observed some intermixed functional connectivity to the CAN in the anterior hippocampus. In higher-resolution (2.6-mm isotropic voxels) and more highly sampled (2,610 min) data, we were able to replicate the observation that the anterior hippocampus is functionally connected to both the DMN and CAN. With more and higher-resolution data, CAN functional connectivity of the anterior hippocampus becomes more readily apparent (*SI Appendix*, Fig. S3). In contrast to the functional border between head/body (DMN + CAN) and tail (PMN + FPN), the network representation of both DMN and CAN in the hippocampal head/body appears to be intermixed. The interdigitated nature of the DMN and CAN in the head/body of the hippocampus even with 2,610 min of 2.6-mm data (MSC06-Rep) suggests that this is not caused by noise or blurring. This could be due to the fact that 1) the DMN proportionally occupies a larger part of the neocortex and/or 2) the CAN is a subnetwork of the DMN (39, 41, 50, 63).

Some have theorized that the CAN mediates the generation of predictions in top-down processing based on learned associations between environmental features (e.g., between objects and their associated contexts) generated from a lifetime of repeating patterns of co-occurrences (49, 56, 63, 64). Therefore, as contextual associations are important for episodic memory and spatial mapping, the anterior hippocampus may be a zone of integration for both the DMN and CAN, utilizing associative knowledge during episodic memory and affective processing. Others have argued that the hippocampus plays a role in binding item information within spatiotemporal contexts (65) and have suggested a role in scene processing (66).

The presence of both CAN and DMN functional connectivity highlights the importance of integrating contextual, social, and affective information in the anterior hippocampus, which is consistent with prior notions of the anterior hippocampus’ interactions with the ventro-medial prefrontal cortex in schema generation (6, 8, 67). This integration is important for constructing and updating schemas (i.e., coherent worldviews) of one’s environment in which the associated episodic memories, spatiotemporal contexts, social cognition and emotions are consistent. The anterior hippocampus-medial parietal cortex circuit may support mental simulations based on autobiographical memory, theory of mind, and self-referential judgments in order to guide expectations and comprehension of the interior life and external environment. A more general description of the anterior circuit’s function may be to support ordinal sequencing in spacetime (68, 69) in the service of the self.

### Posterior Hippocampus to Medial Parietal Cortex Circuit for Goal-Oriented Processing.

The circuit between posterior hippocampus, PMN, and medial parietal parts of the FPN may be part of a system that integrates PMN and FPN functions in order to allow attention-directed memory retrieval. The PMN is deactivated relative to baseline by novel stimuli (48, 53, 70). The PMN is activated by familiar stimuli during explicit novel versus familiar judgments but fails to do so implicitly, without attentional focus on familiarity (70). The PMN’s task-driven activity patterns seem to reflect attention to relevant internal mnemonic representations (48). Meanwhile, the FPN supports executive functions (22) such as directing visual attention. This integration of PMN and FPN in the posterior hippocampus might involve 1) attention to relevant internal memory representations or schemas similar to the current environmental input (48), 2) the retrieval of prior experiences that may be relevant for selecting task-appropriate responses (9, 71), and 3) selecting relevant novel information to update the appropriate internal memory representations (72, 73).

While it is known that the hippocampus is involved in novelty–familiarity discrimination tasks (72, 74), our results suggest that the tail of the hippocampus may be more important for familiarity judgments of recently seen stimuli (3) given the preferential functional connectivity of the tail to the PMN. The PMN may be crucial for long-term memory encoding by directing cognitive resources toward encoding novel information (72, 73).

A notable finding in the present study is that the hippocampal tail was not dominantly functionally connected to occipitotemporal cortex but rather to medial parietal cortex. Prior studies asserted that the posterior hippocampus is functionally connected, perhaps weakly, to visual/perceptual neocortical networks in support of fine, detailed, perceptually rich representations of memories (8, 75). We theorize that attention to and comparison with finer-grained mnemonic representations is necessary to determine whether the current environmental input is familiar or novel without necessitating preferential engagement of visual networks. Across-study differences in the functional connectivity of the posterior hippocampus could be due to 1) use of group-averaged versus individual-specific data and/or 2) the use of winner-take-all methods, which partition the hippocampus by its connectivity to functional networks, not regions of interest.

In addition to being strongly functionally connected to the PMN, the hippocampal tail was also connected to the FPN. Chen et al. found that episodic memory retrieval triggered by recently seen stimuli preferentially activated the PMN and parts of the FPN (53). This PMN–FPN interaction suggests that certain aspects of episodic memory retrieval, such as retrieval of relevant prior experience for task-appropriate responses, requires broader network engagement. Engagement of the PMN and FPN during retrieval of task-relevant prior experience is consistent with the current finding that the tail of the hippocampus is preferentially functionally connected to both the PMN and FPN.

### Comparisons between the Human Hippocampus and Animal Models.

The hippocampus is patterned on its long axis in rodents (ventral–dorsal) and primates (anterior–posterior) (2, 4). In rodents, the majority of the hippocampus (dorsal and midtemporal third) is dedicated to processing visuospatial information while the neocortex largely processes somatosensory inputs and motor outputs (68, 75). With the neocortical expansion in primates, more of the hippocampus is involved in processing nonsensory, associational information (68, 75). Based on our functional parcellation, the more complex functions of the hippocampus (i.e., the self-oriented DMN parcel) are overrepresented compared to the rodent brain, consistent with association cortex expansion. In comparison, the goal- or task-oriented PMN parcel, which is homologous to the visuospatial dorsal hippocampus in rodents, is relatively smaller. The hippocampal tail’s functional connectivity to medial parietal cortex may reflect its conserved role in visuospatial processing (4, 8, 15, 45, 68).

Going forward, studies of human brain function should take into account the dichotomy of hippocampal functional connectivity to cortex, ideally using individual-specific methods. While precision functional mapping may not be feasible in all cases, close approximations could be obtained by anatomically segmenting the hippocampus (Fig. 5) (3).

### Importance of Hippocampal–Neocortical Dialogue across Brain States.

Prior group-averaged human functional connectivity studies, including those reporting anterior–posterior connectivity differences, presumed the hippocampus to be exclusively associated with the DMN. The DMN was originally defined as the brain regions that collectively deactivated during goal-oriented, attention-demanding tasks, independent of the specific task demands (62). Subsequently, the DMN was also shown to be activated by a variety of self-referential, introspective tasks (25, 26). The separation of brain regions into self-oriented (DMN) and task- or goal-oriented (not the DMN) is the first branch point when sorting brain regions according to their fMRI task activity profiles and RSFC data (21). Therefore, awake resting hippocampal function in humans has primarily been linked with the default mode.

The discovery that the tail of the hippocampus is specifically and selectively functionally connected to task-oriented regions thought to be important for controlling attention and memory retrieval suggests that it is specialized for providing hippocampal computations in the task mode. Much larger parts of the hippocampus and cortex seem dedicated to the self- or internally directed default mode (25, 62, 76). Uncoupling of the retrosplenial cortex from other brain regions, including the hippocampus, is associated with disassociation-like behavior (77). This finding highlights the importance of the medial parietal cortex for integrating environmental and sensory information with the egocentric perspective in self-oriented processes. Yet, it appears as if moment-to-moment goal-oriented activity is also dependent on the hippocampus. Differentiable parallel loops between the hippocampus and corresponding medial parietal cortex may be respectively specialized for supporting the self and action, respectively. Thus, the medial parietal cortex may be a bridge for this hippocampal–neocortical dialogue (78, 79) for both self- and goal-oriented processes.

Analyses of high- and low-frequency neural activity in rodents (sharp-wave ripples and theta) and humans (delta-band and infra-slow activity) show that the information flow encoded in high-frequency activity between the hippocampus and cortex reverses its direction during sleep, compared to the awake (resting) state (80–83), primarily in the DMN (79, 83). It is theorized that the direction of low-frequency activity that coordinates this hippocampal–neocortical reciprocal dialogue reflects the cortical-hippocampal state changes between memory encoding (wake) and consolidation (sleep) (83–85).

The previously unrecognized neuroanatomical dichotomization of brain systems for orienting and sequencing in spacetime raises fundamental questions about human memory and the mental processes responsible for our sense of self. Given the functional differentiation of the hippocampal tail from the head/body, does information flow between the tail and neocortex during activity, rest, and sleep follow that of the anterior hippocampus’ DMN parcel? Do anterior and posterior segments still perform the same basic computations coordinated by theta oscillations (68, 86) or slightly different computations that are coordinated via multiple theta generators (87)? Irrespective of the answers to such questions, it seems clear that hippocampal interactions with cortex are critically important across all brain states: sleep, wakeful rest, and action.

## Materials and Methods

### Dataset.

#### Participants and study design.

We employed the publicly available MSC dataset for our analyses (https://openneuro.org/datasets/ds000224). Details of the dataset and processing pipeline have been previously described (34). Here, we describe information about the data and methods that are pertinent to the current study.

The MSC consists of large quantities of fMRI data collected from 10 healthy right-handed adult participants (24 to 34 y; five females), who were recruited from the Washington University in St. Louis community. Participants completed 10 sessions of scanning that were 1.5 h each. Each session consisted of 30 min of resting-state fMRI, in which subjects maintained open-eyed fixation on a white crosshair presented against a black background. The resting-state run was followed by fMRI scans for other tasks: a motor task, semantic judgment task, motion coherence task, and an incidental memory task.

The MSC06-Rep data are part of a publicly available dataset (https://openneuro.org/datasets/ds002766). Details of the dataset and processing pipeline have been previously described (57).

### Structural and fMRI Data Processing.

The processing pipeline was developed for use in individual subjects. The pipeline is available on GitHub (https://github.com/MidnightScanClub/MSCcodebase). We describe the MRI data processing in *SI Appendix*.

### Methods and Statistical Analysis of Hippocampus Functional Connectivity.

#### Manual tracing of the hippocampus.

T1-weighted MRI data initially underwent automated segmentation using Freesurfer version 5.3 followed by manual editing of hippocampal results using ITK-SNAP software by a single highly-experienced rater (D.A.). The hippocampal mask was individual specific. Outlines were inspected and adjusted in the coronal view of the T1-weighted image from posterior to anterior sections. The segmentations were subsequently modified in the axial and sagittal views. The left and right hemispheres were independently outlined. Anatomical boundaries generally followed the approaches of Watson and Thompson, with reference to an anatomic atlas (88–90).

#### Infomap clustering of cortical resting-state networks.

All sessions were concatenated together for each individual before proceeding with the Infomap community detection to 17 cortical networks for each individual MSC subject (33) (*SI Appendix*, Fig. S1). Two medial temporal lobe networks were excluded due to signal bleed from the hippocampus and their poor signal-to-noise ratio.

#### Winner-take-all parcellation of the hippocampus.

We followed previously established winner-take-all approaches for functional parcellation of non-neocortical structures (33, 35) and applied it to the hippocampus. For each given hippocampal voxel, we calculated the average BOLD time course of all cortical vertices greater than 20 mm away from the hippocampus that made up a particular cortical network for all 15 networks. The correlation between every cortical network and the hippocampal voxel were calculated, in which the cortical functional network with the greatest positive correlation strength was declared the winner in the hippocampal voxel. Runner-ups or the second-place winners were identified based on whether the correlation strength was at least 66% of the winning correlation strength (33, 35).

#### Generation of binary masks for the DMN and PMN.

We reran the winner-take-all analysis using just the DMN and PMN networks as potential winner networks to create masks, splitting the hippocampus into two parcels. All subsequent analyses relied on this individualized DMN–PMN parcellation.

#### Hippocampal-cortical functional connectivity maps.

The resulting winner-take-all parcels were used to calculate the functional correlation to all cortical vertices. The mean time course for a parcel was calculated before correlating it with every vertex on the cortex with correlation strengths Fisher z-transformed. The resulting functional connectivity maps were plotted with individualized cortical network boundaries overlaid.

#### Anatomical segmentation of the hippocampus.

The head and body versus the tail of the hippocampus were anatomically defined by landmarks outlined in Daugherty et al. (59) The landmarks were identified in the session-averaged T1-weighted structural image for each individual to identify the coronal slice in which the fornix appears posteriorly to the thalamus (59). Identification of the coronal slice was double-checked for accuracy by a neuroradiologist (J.S.S.). All hippocampal voxels posterior to said coronal slice was considered the tail of the hippocampus, whereas all voxels anteriorly were considered the head/body of the hippocampus. Anatomical segmentation of the hippocampus can also be achieved using the percentage-based method (3).

#### Task deactivations.

After standard fMRI preprocessing as outlined in the section “Functional MRI (fMRI) preprocessing” in the *SI Appendix*, task fMRI data were processed as previously described (34, 91). We used a pair of mixed block-/event-related design tasks which comprised language and perceptual task trials in order to model task-based deactivations (34, 91).

Task fMRI data were entered in a General Linear Model (GLM) separately for each session from each individual using in-house software (FIDL) (92). The mixed design tasks were modeled jointly in a single GLM with separate event regressors for onset and offset cues from each task, trials in each task, and a sustained block regressor for the task period. Event regressors were modeled using a finite impulse response approach consisting of delta functions at each of eight time points, allowing for the more complete modeling of different hemodynamic response function shapes (93). Deactivations were identified using a contrast of the third and fourth time points from all conditions in the mixed design tasks (against an implicit unmodeled baseline).

## Supplementary Material

Supplementary File

## Data Availability

This study used previously published datasets (34, 57), which are available on OpenNeuro. The MSC dataset (94) is publicly available on OpenNeuro (https://openneuro.org/datasets/ds000224) as is the MSC06-Rep data (95) (https://openneuro.org/datasets/ds002766). The processing pipeline is publicly available on GitHub (https://github.com/MidnightScanClub/MSCcodebase).
