# Supplementary material for: Parallel hippocampal-parietal circuits for self- and goal-oriented processing
Source: Proc Natl Acad Sci U S A. 2021 Aug 17;118(34):e2101743118. doi: 10.1073/pnas.2101743118 (PMC8403906; doi:10.1073/pnas.2101743118)
Supplement: Supplementary File [file pnas.2101743118.sapp.pdf]

PNAS
www.pnas.org

Supplementary Information for
Parallel Hippocampal-Parietal Circuits for Self- and Goal-oriented Processing

Annie Zheng*[1], David F. Montez[1], Scott Marek[2], Adrian W. Gilmore[3], Dillan J. Newbold[1], Timothy O. Laumann[2], Benjamin P. Kay[1], Nicole A. Seider[1], Andrew N. Van[1], Jacqueline M. Hampton[1,2], Dimitrios Alexopolous[1], Bradley L. Schlaggar[4,5,6], Chad M. Sylvester[2], Deanna J. Greene[7], Joshua S. Shimony[8], Steven M. Nelson[9,10], Gagan S. Wig[11,12], Caterina Gratton[13,14], Kathleen B. McDermott[8,15], Marcus E. Raichle[1,8], Evan M. Gordon[8], Nico U.F. Dosenbach**[1,8,16,17,18]

1. Department of Neurology, Washington University School of Medicine, St. Louis, MO, USA
2. Department of Psychiatry, Washington University School of Medicine, St. Louis, MO, USA
3. Section on Cognitive Neuropsychology, Laboratory of Brain and Cognition, National Institute of Mental Health, National Institutes of Health, Bethesda, MD, USA
4. Kennedy Krieger Institute, Baltimore, MD, USA
5. Department of Neurology, Johns Hopkins University School of Medicine, Baltimore, MD, USA
6. Department of Pediatrics, Johns Hopkins University School of Medicine, Baltimore, MD, USA
7. Department of Cognitive Science, University of California San Diego, San Diego, CA, USA
8. Mallinckrodt Institute of Radiology, Washington University School of Medicine, St. Louis, MO, USA
9. Department of Pediatrics, University of Minnesota, Minneapolis, MN, USA
10. Masonic Institute for the Developing Brain, University of Minnesota, Minneapolis, MN, USA
11. Center for Vital Longevity, School of Behavioral and Brain Sciences, University of Texas at Dallas, Dallas, TX, USA
12. Department of Psychology and Neuroscience, Baylor University, Waco, TX, USA
13. Department of Psychology, Northwestern University, Evanston, IL, USA
14. Department of Neurology, Northwestern University, Evanston, IL, USA
15. Department of Psychological and Brain Sciences, Washington University in St. Louis, St. Louis, MO, USA
16. Department of Biomedical Engineering, Washington University in St. Louis, St. Louis, MO, USA
17. Department of Pediatrics, Washington University School of Medicine, St. Louis, MO, USA
18. Program in Occupational Therapy, Washington University School of Medicine, St. Louis, MO, USA

*Lead Contact: Annie Zheng
Email: azheng@wustl.edu

**Alternate Contact: Nico Dosenbach
Email: ndosenbach@wustl.edu

This PDF file includes:

Supplementary Methods
Figures S1 to S13
Tables S1
SI References

METHODS: STRUCTURAL & FUNCTIONAL MRI DATA PROCESSING

The preprocessing stream, individual-specific cortical surface generation, mapping of BOLD data to individual-specific cortical surfaces, and Infomap-derived individual-specific for the MSC rs-fMRI data have all been previously described in published articles (*1*–*3*). Here, we describe the relevant details.

<u>MRI</u> <u>image</u> <u>acquisition</u>

Participants were scanned on a Siemens TRIO 3T with whole brain coverage, which allowed for the examination of the hippocampus' functional connectivity to all of cortex, not just to cortex proximate to the hippocampus. Structural images included four T1-weighted scans (TE = 3.74ms, TR = 2400ms, TI = 1000ms, flip angle = 8°, 0.8mm isotropic voxels, 224 sagittal slices) and four T2-weighted images (TE = 479ms, TR = 3200ms, 0.8mm isotropic voxels, 224 sagittal, 224).

Functional images included 300 minutes of eyes-open resting state fMRI BOLD data (10 sessions x 30min/session) and 350 minutes total of task fMRI BOLD data using a gradient-echo EPI BOLD sequence (TE = 27ms, TR = 2.2s, flip angle = 90°, 4mm isotropic voxels, 36 axial slices). Gradient echo field map images (one per session) were acquired with the same parameters. See Gordon *et al.* for more details (*2*).

One participant (MSC06) underwent an additional 87 imaging sessions on a Siemens Prisma 3T MRI scanner, consisting of fMRI scans with higher resolution (gradient-echo EPI BOLD sequence: multiband factor 4, TE = 33ms, TR = 1.1 s, flip angle = 84°, 2.6mm isotropic voxels, 56 axial slices). These additional resting state fMRI scans were conducted as part of another study independent of the MSC data collection (*4*), but are labeled as MSC06-Rep in this article. See (*4*) for more details on image acquisition parameters and procedures. We used MSC06-Rep to validate the results from the original 10 MSC subjects, and as a way to assess the effects of spatial resolution.

<u>Structural</u> <u>MRI</u>

Cortical surfaces were generated according to procedures described in Laumann *et al.* (*5*). Each participant's averaged T1-weighted image was run through FreeSurfer v5.3's recon-all processing pipeline to create the anatomical surface, which was double-checked and manually edited using Freeviewer. Surfaces were then registered into fs_LR_32k surface space with the Multi-modal Surface Matching algorithm (*6*).

<u>Functional</u> <u>MRI</u> <u>(fMRI)</u> <u>preprocessing</u>

All fMRI data were preprocessed to maximize cross-session registration, which involved slice-time correction, intensity normalization, and within-run head motion correction. The functional MRI data were registered to Talairach atlas space using the subject-specific averaged T2-weighted and T1-weighted structural image. We used the mean field map to apply a distortion correction for each participant before resampling into 2mm isotropic resolution. These steps were combined into a single interpolation using FSL's applywarp tool (*7*).

<u>Resting</u> <u>state</u> <u>fMRI</u> <u>(rs-fMRI)</u> <u>data</u> <u>preprocessing</u>

We further preprocessed the rs-fMRI data to reduce spurious effects that are likely unrelated to neural activity using a motion censoring procedure described in Power *et al.* (*8*) in line with current best practices for reducing motion artifacts (*9*). Motion-contaminated frames were identified based on a framewise displacement (FD) > 0.2mm or a temporal derivative of the root mean squared variance over voxels (DVARS) > 5.36. Two subjects (MSC03, MSC10) required additional correction for artifactual high-frequency motion in the phase encoding direction (anterior-posterior) as previously described (*2*, *3*).

After motion censoring, the data were further preprocessed with the following additional steps: (1) demeaning and detrending, (2) interpolating censored frames with least-squares spectral estimation, (3) temporal band-pass filtering (0.005 Hz < f < 0.01 Hz), and (4) multiple regression of nuisance variables, which include the global signal, principle components of ventricular and white matter signals (described below in ‘‘Component-based nuisance regression’’), and motion estimates derived from the Volterra expansion (*10*), applied in a single step to the filtered, interpolated BOLD time series. Finally, censored volumes were removed from the data for all subsequent analyses. Application of the temporal masks resulted in retention of 5704 ± 1548 volumes per subject (range of 2691-7530) or ~209 ± 57 min.

For MSC06's additional 2.6mm resolution data, the data were processed in the same manner described above. However, (1) FD measurements were corrected for artifactual high-frequency motion in the phase encoding direction, (2) the FD threshold for motion censoring was 0.1mm, and (3) the DVARS threshold for motion censoring was 6.
The cortical data were then registered to the surface (see above "Structural MRI"). The cortical surface data and volumetric subcortical and cerebellar data were combined into CIFTI data format using the Connectome Workbench toolbox (*11*). Two independent atlas spaces are used: (1) a volumetric atlas (Talairach) and (2) a surface atlas (fs_LR_32k) to allow for comparison across subjects for both the subcortical volumes and cortical surfaces.

Voxels in the cerebellum and the subcortical structures were derived from the FreeSurfer segmentation of each subject's native averaged T1-weighted image and manually edited by expert neuroanatomists to ensure utmost accuracy in grey matter segmentation. These were then transformed into Talairach atlas space. Finally, the cortical surface functional data were smoothed (2D geodesic, Gaussian kernel, σ = 2.55mm). Due to the small size of subcortical structures, we did not spatially smooth data within the subcortical volume and we up-sampled the fully processed data to 2mm isotropic voxels.

Component-based nuisance regression

The temporally filtered BOLD time series underwent a component-based nuisance regression approach (*1*, *12*, *13*). We built nuisance regressors based off of individual-specific white matter and ventricle masks, which were segmented by FreeSurfer (*14*), spatially resampled, and registered to the fMRI data. As voxels at the edges of the brain are highly susceptible to motion and CSF artifacts (*15*, *16*), we created another nuisance mask specifically for the extra-axial compartment by thresholding the temporal standard deviation image (SD_t>2.5%), excluding a dilated whole brain mask (*17*, *18*).

We applied dimensionality reduction to the voxel-wise nuisance time series as outlined in CompCor (*18*). However, the number of retained regressors was determined for each noise compartment by orthogonalizing the covariance matrix and retaining components ordered by decreasing eigenvalue up to a condition number of 30 ($\lambda_{max}/\lambda_{min} > 30$). The columns of the design matrix *X* comprised the retained components across all compartments, the global signal and its first derivative, and the six motion correction time series. Since the columns of the design matrix *X* may exhibit collinearity, we applied a second level SVD to XX^T to overcome potential rank-deficiency in the design matrix. This imposed an upper limit of 250 on the condition number. The regressors were applied in a single step to the filtered, interpolated BOLD time series.

Distance-based regression of adjacent grey matter cortical tissue

The hippocampus is in close proximity to cortex, which results in signal contamination and spurious correlations between hippocampal voxels and adjacent grey matter vertices. Therefore, we quantified the Euclidean distance between every hippocampal voxel and every grey matter vertex. For each hippocampal voxel, we then generated the average BOLD signal time course of adjacent gray matter vertices within 20 mm and removed it by regression. This follows similar strategies taken in previous work on subcortical functional connectivity (*1*, *12*, *19*).

FIGURES

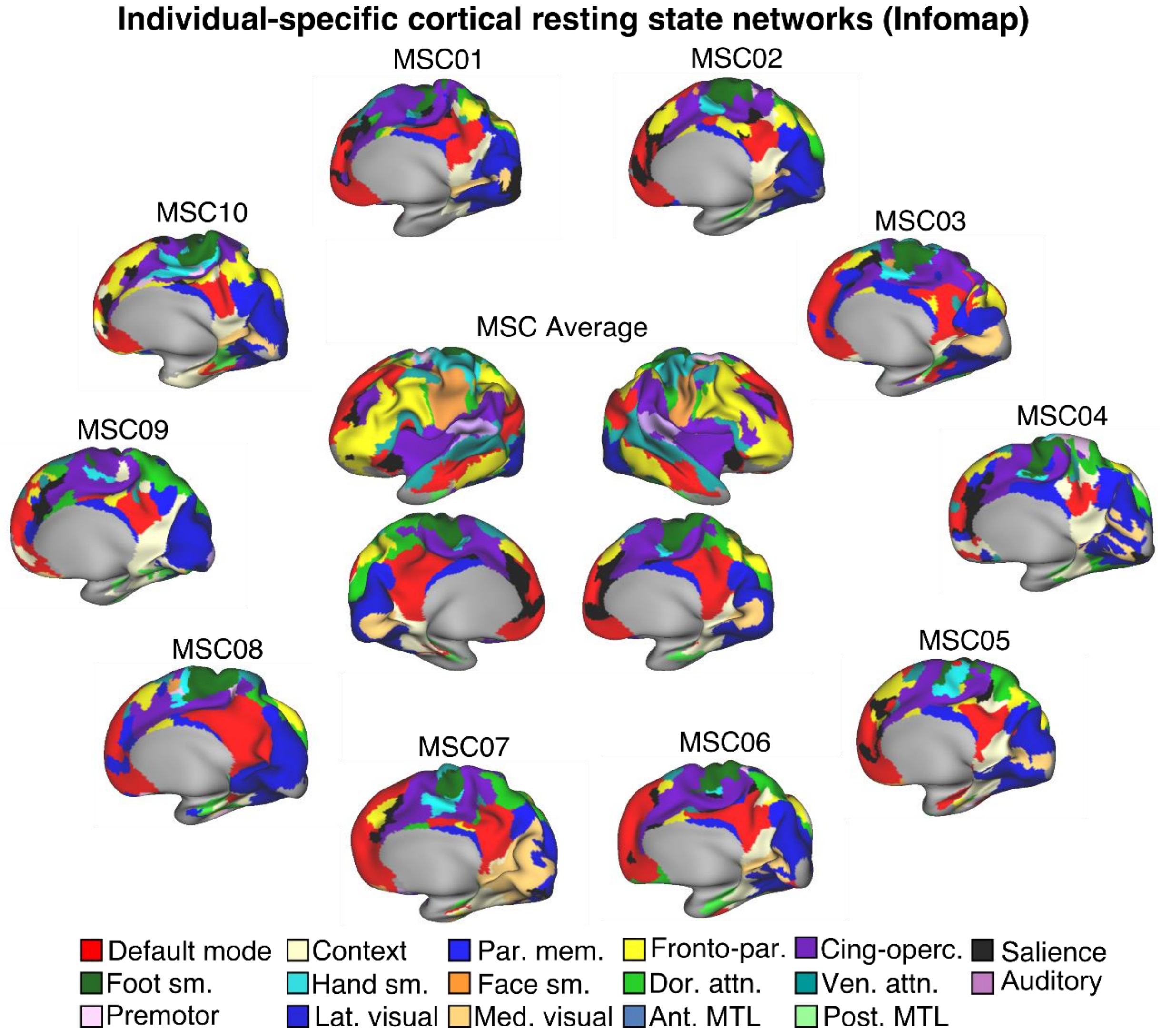

Figure S1. individual-Specific Cortical Resting State Networks. Using the Infomap community detection algorithm, cortical resting state networks were defined for each individual MSC subject as well as the MSC Average. These individually-specific networks were then used for the winner-take-all parcellation of the hippocampus.

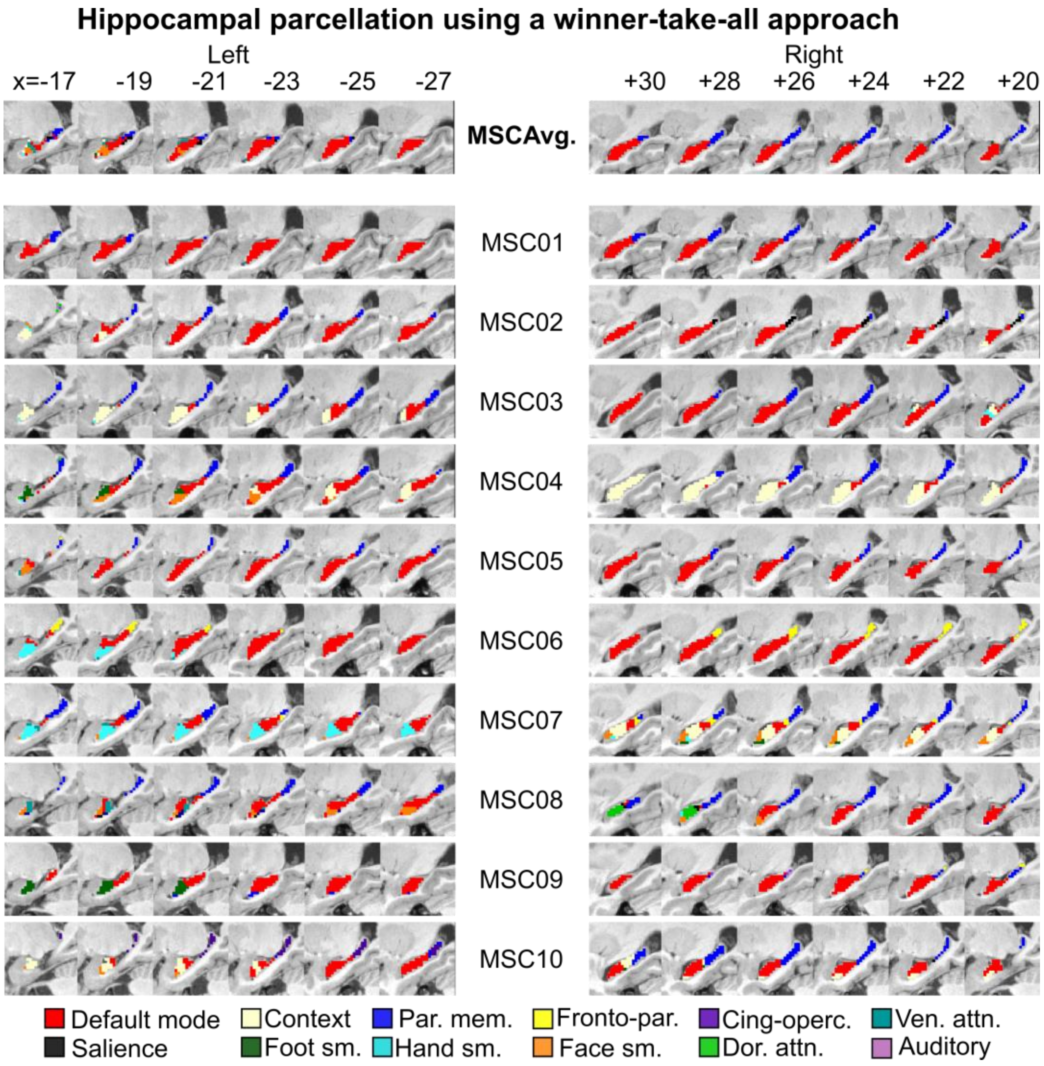

Figure S2. Hippocampal Parcellation Using a Winner-Take-All Approach. Winner-Take-All parcellation of the hippocampus based on its functional connectivity to all cortical networks for the MSC Average and each subject. Parasagittal slices are shown.

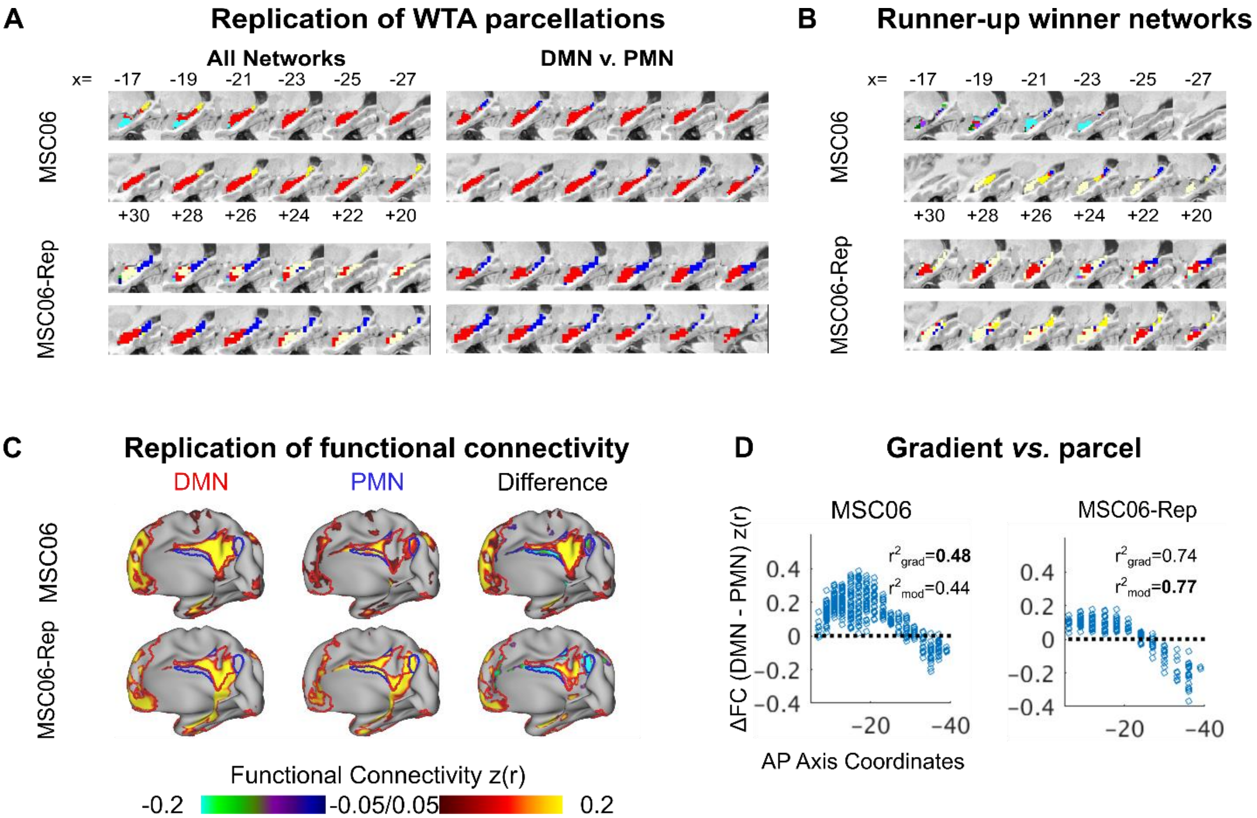

Figure S3. Replication of Winner-Take-All Parcellation in Higher-Resolution, Higher-Sampled Resting State fMRI Data from MSC06. (A) Top panel shows the original network localization in the hippocampus for MSC06 using all potential winner networks (left) and two networks (DMN *vs.* PMN) as reference. The bottom panel (MSC06-Rep) shows a replication of MSC06's winner-take-all parcellation using higher-resolution data (2.6mm isotropic voxels). **(B)** Displayed are the runner-up or second-place winning networks of the winner-take-all parcellation of the hippocampus for MSC06 and MSC06-Rep. **(C)** Functional connectivity seed maps of right hippocampal DMN and PMN parcels replicate across MSC06 and MSC06-Rep. **(D)** The amount of variance in functional connectivity differences explained by the gradient (grad) and parcel (par) models are noted, suggesting equal variance explained, and thereby, both a gradient and parcel organization. Functional connectivity values z(r) are Fisher z-transformed.

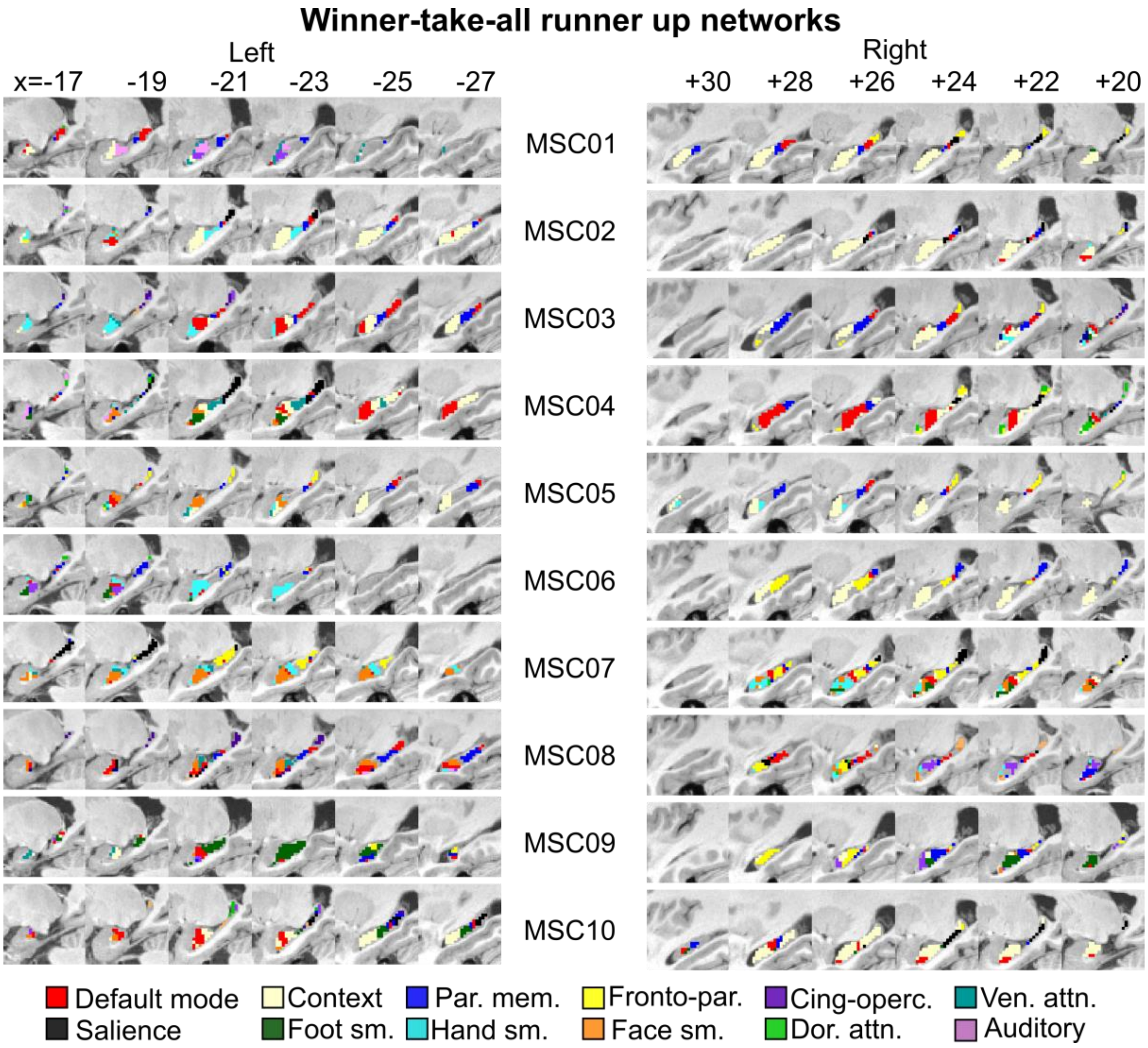

Figure S4. Runner-up Winner-Take-All Parcellation of the Hippocampus. Displayed are the runner-up or second-place winning networks of the winner-take-all parcellation of the hippocampus for all 10 MSC subjects. Not all hippocampal voxels were categorized as having multiple network connectivity; hence, some voxels are not colored. Voxels that were categorized as having runner-up network connectivity was based on if the runner-up correlation strength was at least 66.7% of the winning correlation following previously-established procedures (*34*, *36*).

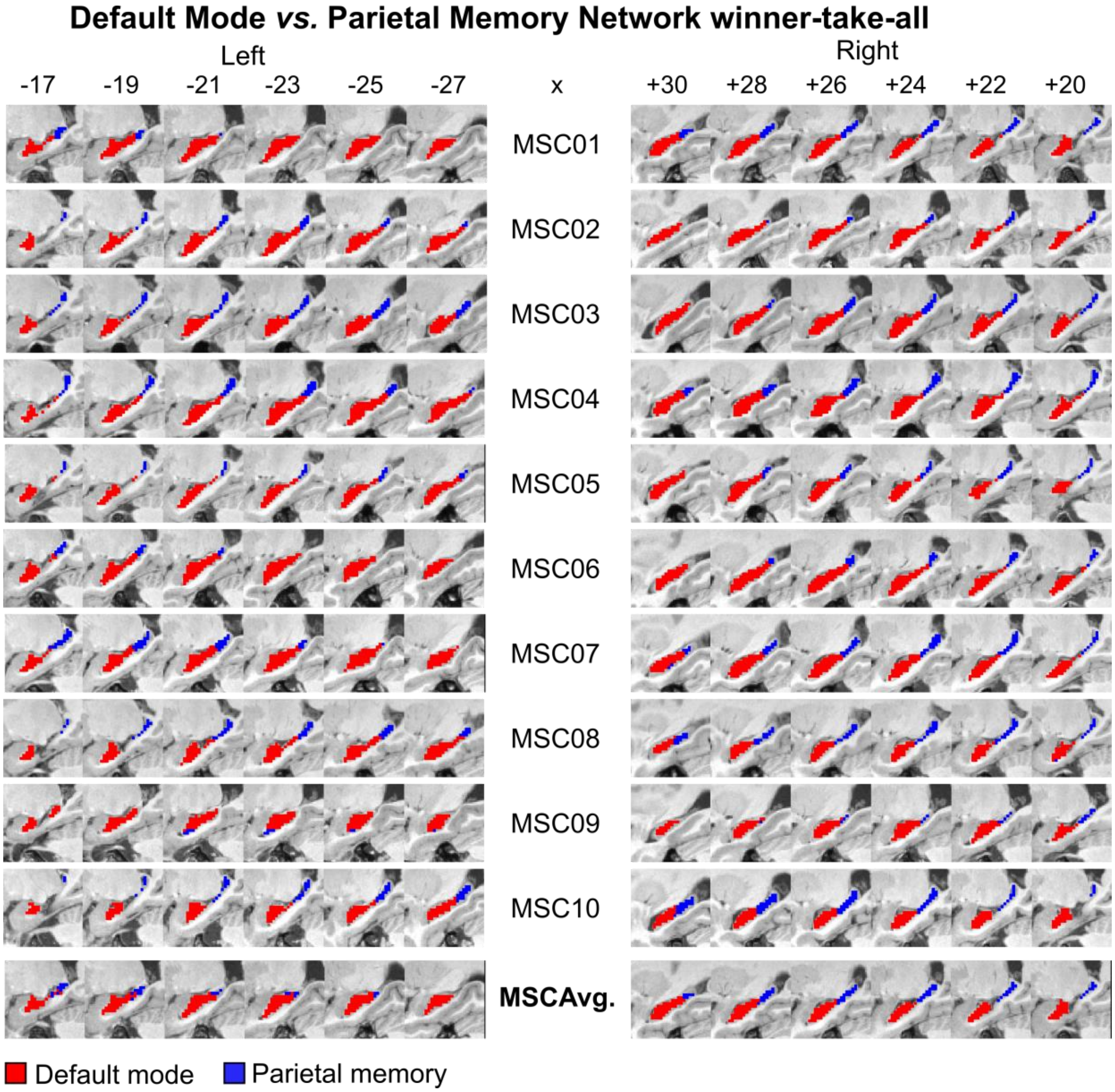

Figure S5. Hippocampal Parcellation Using a Two-Network Winner-Take-All Approach. Anterior-posterior dichotomy in default mode network (DMN, red) and parietal memory network (PMN, blue) representations in the left and right hippocampus for the MSC Average and each subject. Parasagittal slices shown.

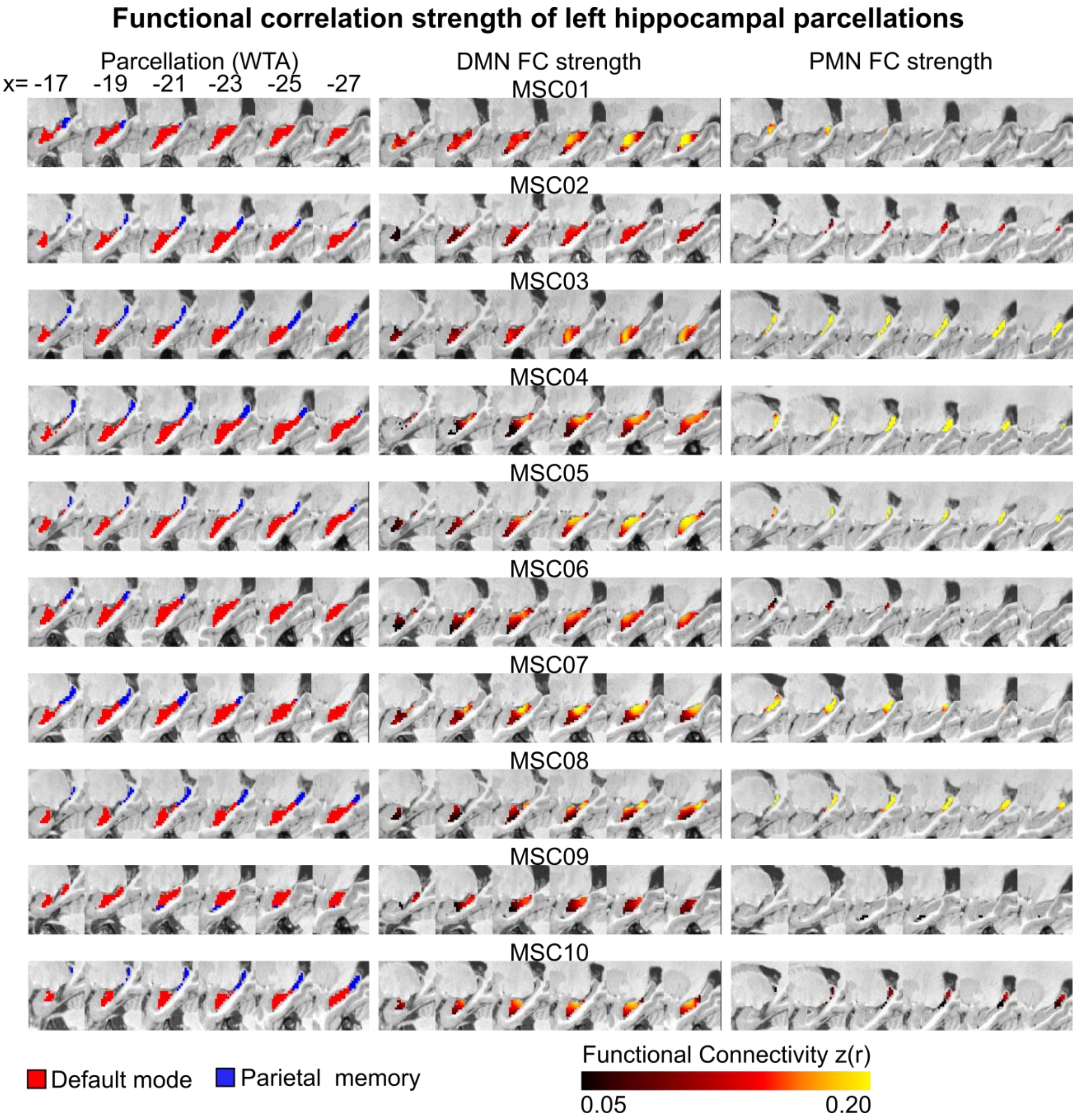

Figure S6. Functional Connectivity Strength of Winner-Take-All Parcellations in the Left Hippocampus. The 2-network (DMN vs. PMN) winner-take-all parcellation (left) of the left hippocampus is shown side by side with the DMN (center) and PMN (right) Fisher z-transformed functional correlation strength [z(r)] to their respective winner networks.

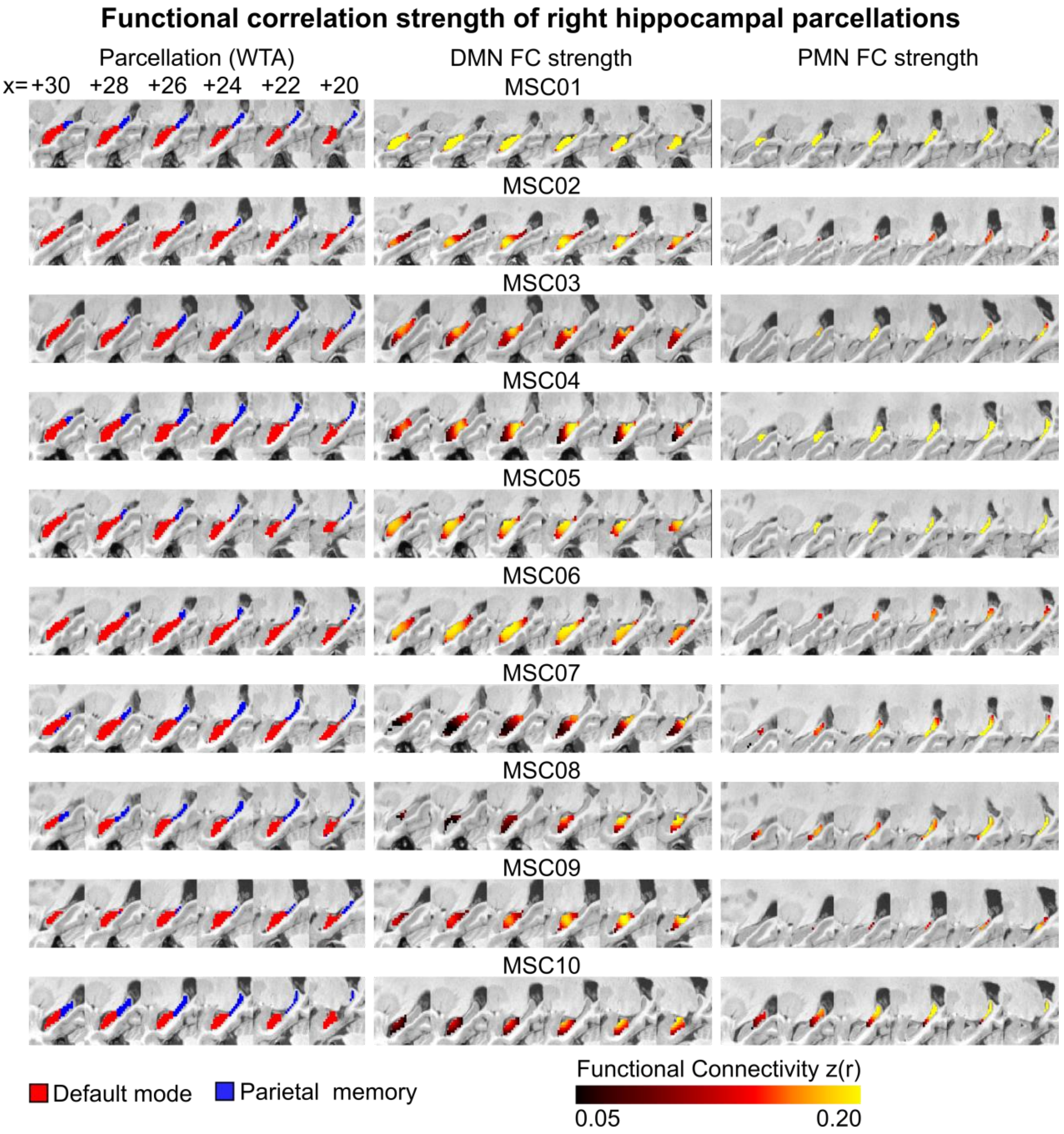

Figure S7. Functional Connectivity Strength of Winner-Take-All Parcellations in the Right Hippocampus. The 2-network (DMN vs. PMN) winner-take-all parcellation (left) of the right hippocampus is shown side by side with the DMN (center) and PMN (right) Fisher z-transformed functional correlation strength [z(r)] to their respective winner networks.

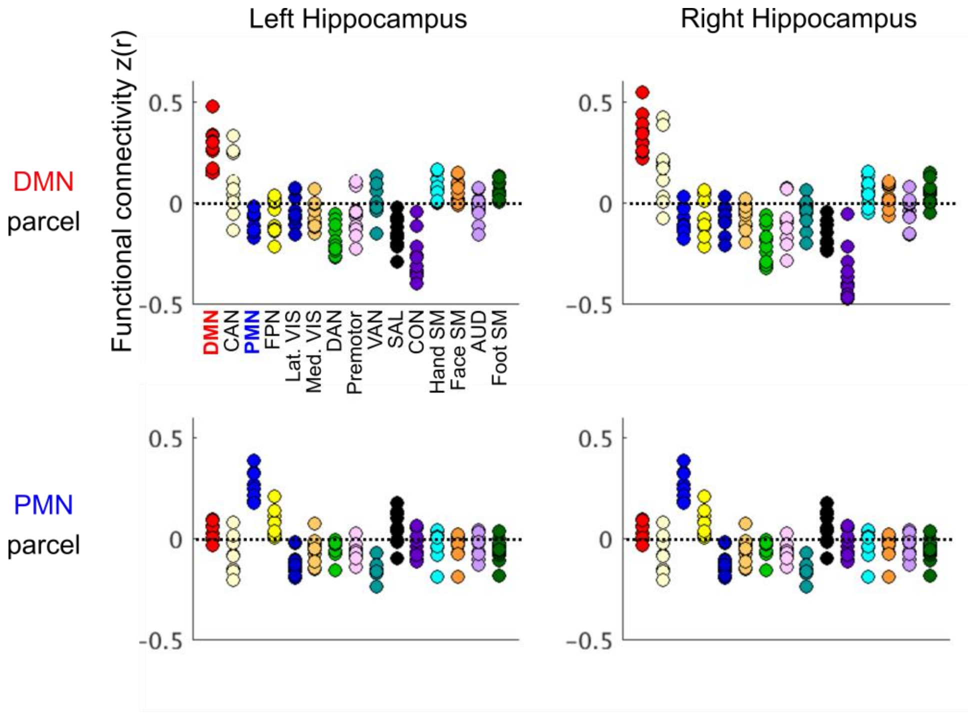

Figure S8. Mean Functional Connectivity of Winner-Take-All Parcels to Cortical Resting State Networks. The DMN (top) and PMN (bottom) parcels' mean functional connectivity to all cortical resting state networks for the left and right hippocampus for each MSC subject. In addition to the parcels' Fisher z-transformed functional connectivity z(r) to its winning network.

Subject	Gradient	Parcel
MSC01	0.063	0.046
MSC02	**0.183**	0.018
MSC03	**0.108**	0.065
MSC04	0.003	**0.279**
MSC05	0.034	**0.119**
MSC06	**0.118**	0.076
MSC07	**0.095**	0.054
MSC08	0.003	**0.142**
MSC09	0.047	**0.056**
MSC10	**0.075**	0.011
MSC06-Rep	0.061	**0.090**

Table S1. Amount of Variance in Hippocampal Functional Connectivity Explained by Gradient and Parcel Factors. ANCOVA testing both the gradient (AP axis) and parcel factors head-to-head. The table depicts the amount of variance explained in functional connectivity difference (DMN - PMN) by one factor, while accounting for the other for all MSC subjects and MSC06-Rep. The amount of variance explained by each factor alone is shown in **Figure 4** for the MSC subjects and in **Figure S3D** for MSC06-Rep.

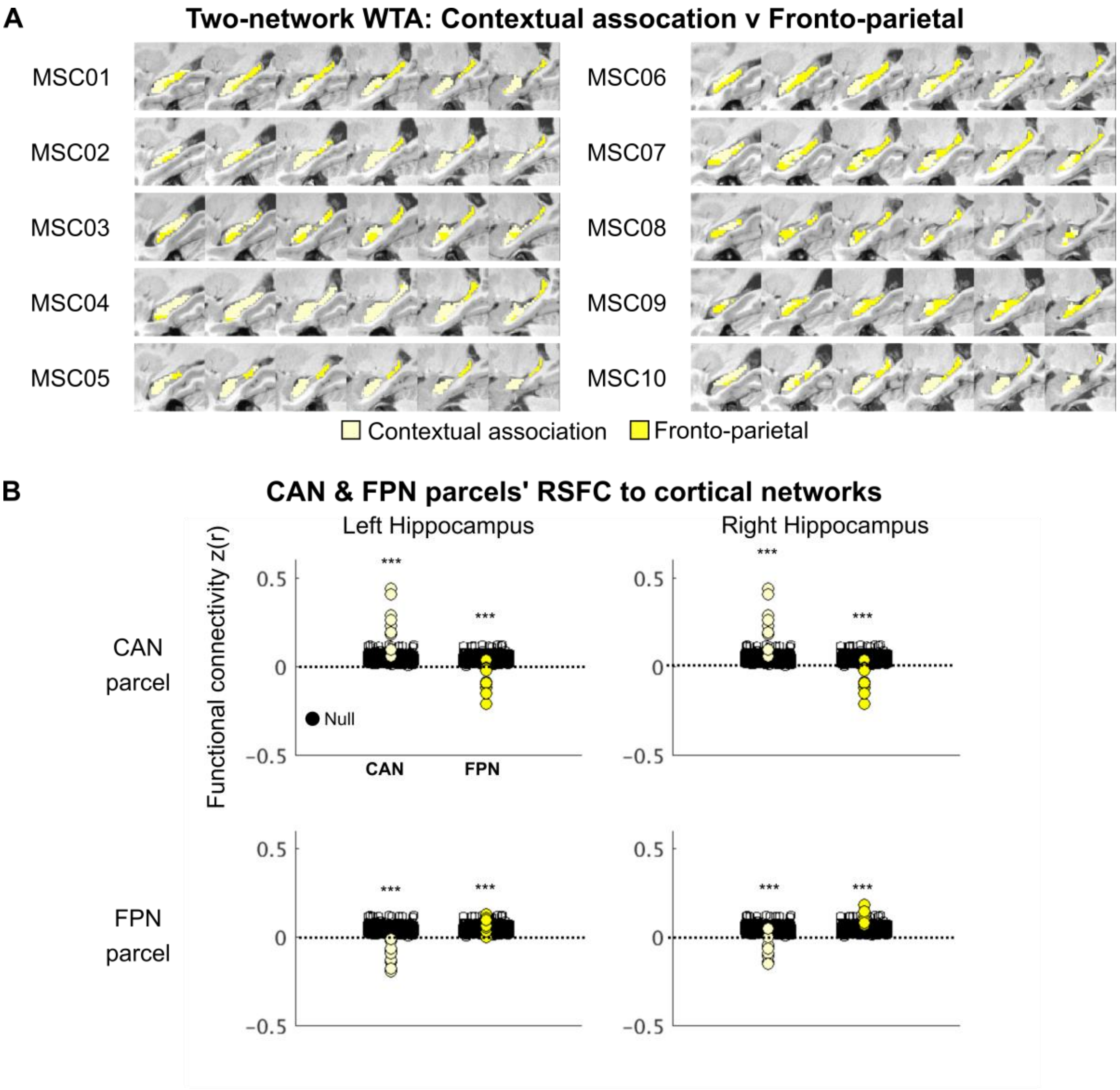

Figure S9. Significance Testing of Two-Network (CAN & FPN) Winner-Take-All Parcellation of the Hippocampus. We first defined the **(A)** winner-take-all parcellation of the hippocampus using the CAN and FPN, which demonstrates an anterior-posterior axis of organization. The anterior hippocampus is connected to the CAN and the posterior to the FPN. Using the defined CAN and FPN parcels, we found that **(B)** the parcels' Fisher z-transformed functional connectivity z(r) to their winner networks is significantly different from a participant-specific null distribution.

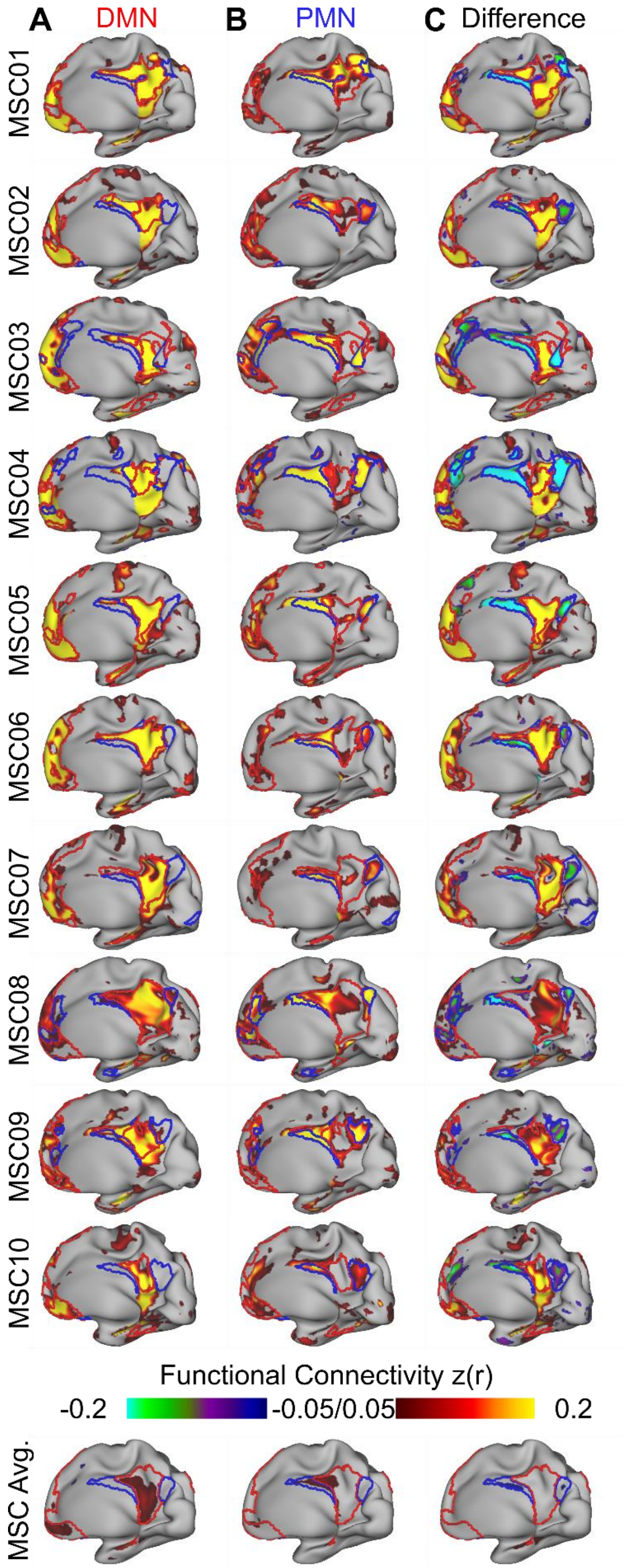

Figure S10. Functional Connectivity of Individual-Specific Hippocampal DMN and PMN parcels for right hippocampus. Right hemisphere is shown. **(A)** The functional connectivity patterns of the anterior, default mode network (DMN) and **(B)** the posterior, parietal memory network (PMN) parcels in the hippocampus are shown. **(C)** The difference maps of functional connections for the right hippocampus. The warm colors in the color scale represent greater DMN correlation and the cool colors represent greater PMN correlation. Functional connectivity values z(r) are Fisher z-transformed.

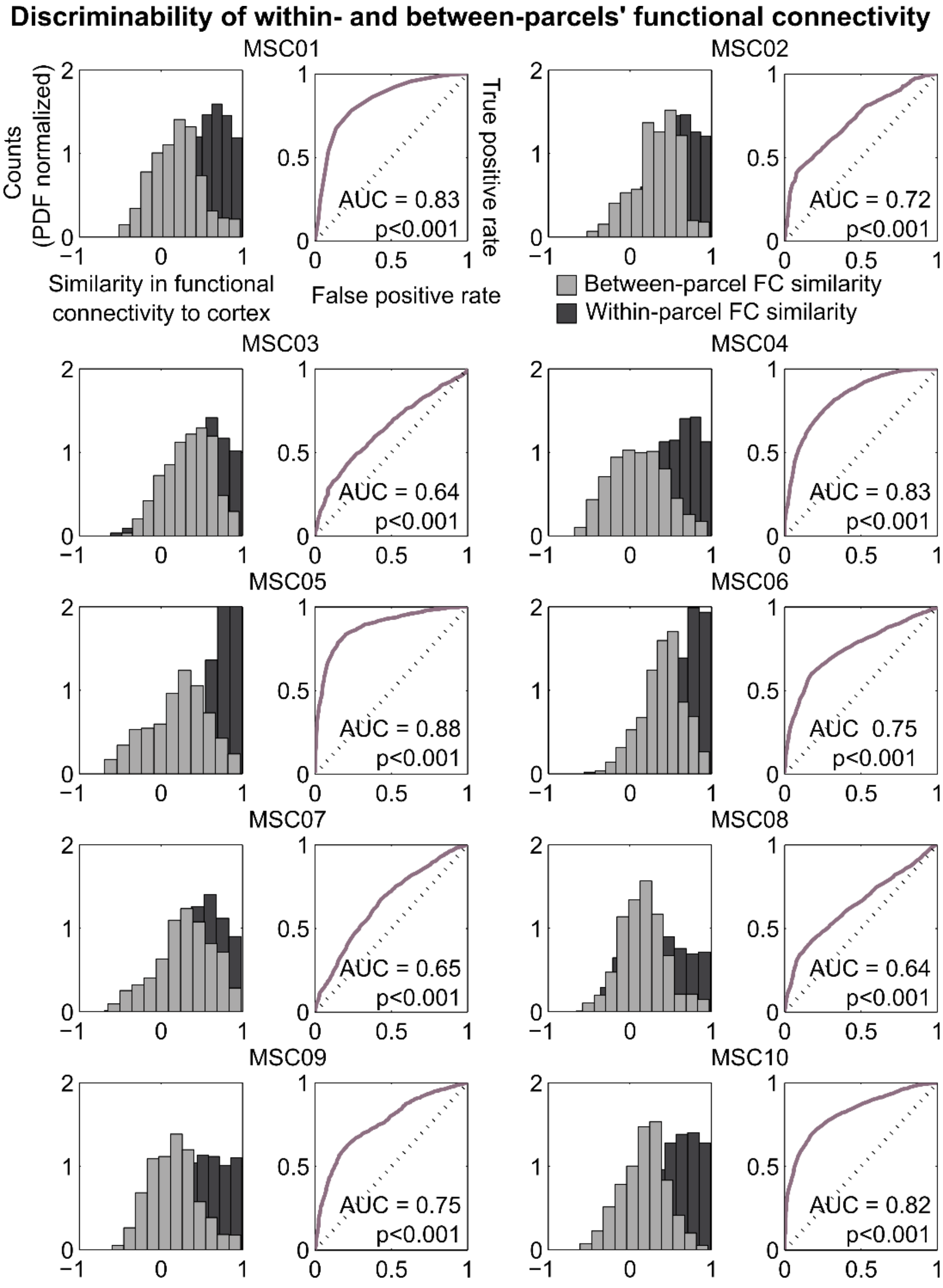

Figure S11. Discriminability of Within- and Between-parcels' Functional Connectivity to the Cortex. The distribution of similarity in functional connectivity seed maps between pairs of voxels that are within the same hippocampal parcel *vs.* between different hippocampal parcels are discriminable as defined by a receiver-operator characteristic (ROC) curve. The area under the curve (AUC) represents the probability that an ideal observer would be able to adjudicate between these two distributions.

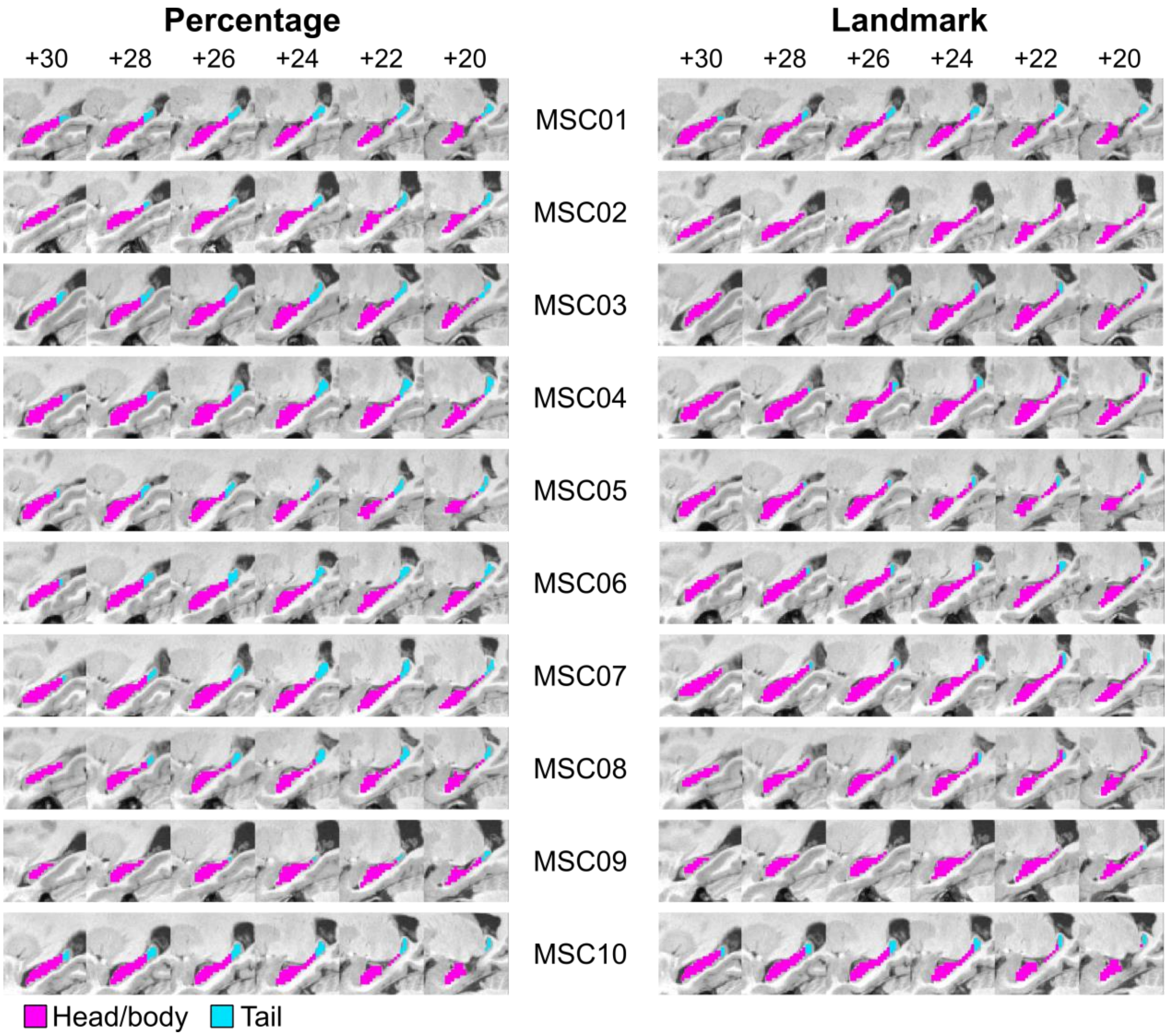

Figure S12. Anatomical Segmentation of the Hippocampus into Head/Body and Tail. We used two methods for an anatomical segmentation: a percentage-based approach (80%-20% for head/body *vs.* tail) as well as anatomical landmarks to segment the hippocampal body from the tail for each individual according to their unique hippocampal anatomy. Anatomical segmentations for all subjects shown for the right hippocampus, demonstrating a high degree of agreement (Dice coefficient median: 0.92, range: 0.85-0.96). Anatomical segmentations were then used to generate spatial correlation maps with the cortex in **Figure 5**.

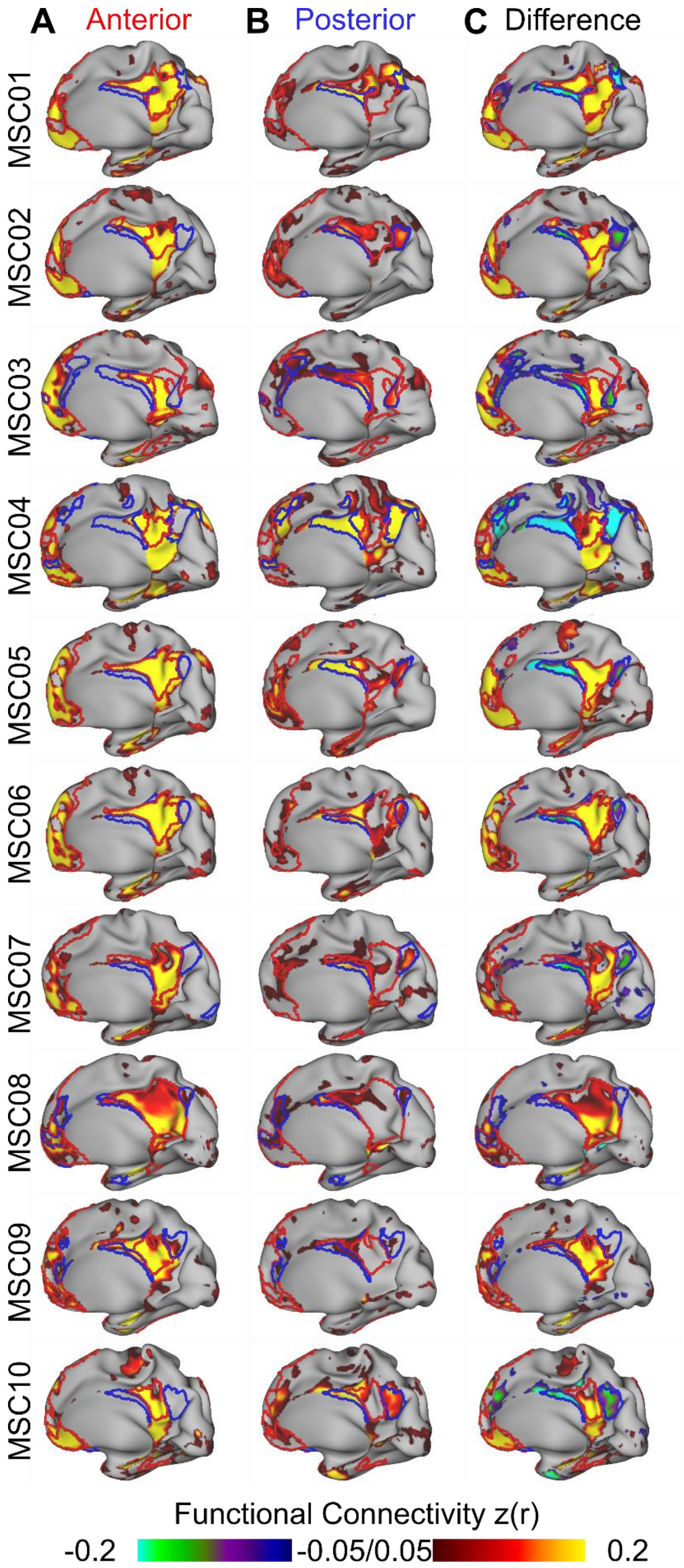

Figure S13. Functional Connectivity of Anatomically Defined Hippocampal Segments (Head/body vs. Tail). Functional connectivity seed map for **(A)** head/body parcel, **(B)** tail and **(C)** the difference between the two for all subjects (head/body − tail). Functional connectivity maps for anatomical parcels (from the landmark-based approach) recapitulate winner-take-all-derived seed maps. Functional connectivity values z(r) are Fisher z-transformed.

SI REFERENCES

1. S. Marek *et al.*, Spatial and Temporal Organization of the Individual Human Cerebellum. *Neuron*. **100** (2018), doi:https://doi.org/10.1016/j.neuron.2018.10.010.
2. E. M. Gordon *et al.*, Precision Functional Mapping of Individual Human Brains. *Neuron*. **95**, 791-807.e7 (2017).
3. C. Gratton *et al.*, Functional Brain Networks Are Dominated by Stable Group and Individual Factors, Not Cognitive or Daily Variation. *Neuron*, 439–452 (2018).
4. D. J. Newbold *et al.*, Plasticity and Spontaneous Activity Pulses in Disused Human Brain Circuits. *Neuron*. **107**, 580-589.e6 (2020).
5. T. O. Laumann *et al.*, Functional System and Areal Organization of a Highly Sampled Individual Human Brain. *Neuron*. **87**, 658–671 (2015).
6. M. F. Glasser *et al.*, A multi-modal parcellation of human cerebral cortex. *Nature*. **536**, 171–178 (2016).
7. S. M. Smith *et al.*, Advances in functional and structural MR image analysis and implementation as FSL. *Neuroimage*. **23**, 208–219 (2004).
8. J. D. Power *et al.*, Methods to detect, characterize, and remove motion artifact in resting state fMRI. *Neuroimage*. **84**, 1–45 (2014).
9. R. Ciric *et al.*, Benchmarking of participant-level confound regression strategies for the control of motion artifact in studies of functional connectivity. *Neuroimage*. **154**, 174–187 (2017).
10. K. J. Friston, S. Williams, R. Howard, R. S. J. Frackowiak, R. Turner, Movement-related effects in fMRI time-series. *Magn. Reson. Med.* **35**, 346–355 (1996).
11. D. S. Marcus *et al.*, Informatics and Data Mining Tools and Strategies for the Human Connectome Project. *Front. Neuroinform.* **5**, 1–12 (2011).
12. D. J. Greene *et al.*, Integrative and Network-Specific Connectivity of the Basal Ganglia and Thalamus Defined in Individuals. *Neuron*. **105**, 1–17 (2020).
13. R. V. Raut, A. Mitra, A. Z. Snyder, M. E. Raichle, On time delay estimation and sampling error in resting-state fMRI. *Neuroimage*. **194**, 211–227 (2019).
14. B. Fischl, FreeSurfer. *Neuroimage*. **62**, 774–781 (2012).
15. C. G. Yan *et al.*, A comprehensive assessment of regional variation in the impact of head micromovements on functional connectomics. *Neuroimage*. **76**, 183–201 (2013).
16. T. D. Satterthwaite *et al.*, An improved framework for confound regression and filtering for control of motion artifact in the preprocessing of resting-state functional connectivity data. *Neuroimage*. **64**, 240–256 (2013).
17. R. Patriat, E. K. Molloy, R. M. Birn, Using Edge Voxel Information to Improve Motion Regression for rs-fMRI Connectivity Studies. *Brain Connect.* **5**, 582–595 (2015).
18. Y. Behzadi, K. Restom, J. Liau, T. T. Liu, A component based noise correction method (CompCor) for BOLD and perfusion based fMRI. *Neuroimage*. **37**, 90–101 (2007).
19. D. J. Greene *et al.*, Developmental Changes in the Organization of Functional Connections between the Basal Ganglia and Cerebral Cortex. *J. Neurosci.* **34**, 5842–5854 (2014).
